# Dual efficacy-toxicity of *Chelidonii Herba* in chronic obstructive pulmonary disease: Integrated network pharmacology, immune profiling and molecular docking

**DOI:** 10.1371/journal.pone.0332750

**Published:** 2025-09-23

**Authors:** Guoliang Chen, Tianqing Wang

**Affiliations:** 1 Liaoning University of Traditional Chinese Medicine, Shenyang, Liaoning, China; 2 The Second Affiliated Hospital of Liaoning University of Traditional Chinese Medicine, Shenyang, Liaoning, China; Columbia University Irving Medical Center, UNITED STATES OF AMERICA

## Abstract

**Objective:**

To investigate the pharmacodynamic material basis, multi-target mechanisms of *Chelidonii Herba* in treating chronic obstructive pulmonary disease (COPD), and its hepatotoxicity pathways using network pharmacology, network toxicology, and molecular docking.

**Methods:**

Active components and targets of *Chelidonii Herba* were screened via Traditional Chinese Medicine Systems Pharmacology (TCMSP), SwissTargetPrediction (STP), and PharmMapper databases. COPD and hepatotoxicity targets were obtained from GeneCards and OMIM. Venn diagrams identified shared targets. Protein-protein interaction (PPI) networks were constructed using STRING, with core targets filtered via CytoNCA. Gene Ontology (GO) and Kyoto Encyclopedia of Genes and Genomes (KEGG) enrichment analyses were performed in Metascape. Molecular docking was validated by AutoDock Vina, and immune infiltration was analyzed using the GSE55962 dataset.

**Results:**

Twenty active components and 108 potential targets of *Chelidonii Herba* were identified. Eighty shared targets intersected with COPD, and 96 with hepatotoxicity. Seven core targets for COPD treatment (CASP3, PPARG, PTGS2, CDK2, ALB, HSP90AA1, ESR1) and hepatotoxicity (PPARG, ESR1, CASP3, PTGS2, ESR2, CALM3, ALB) were determined. KEGG enrichment revealed COPD mechanisms involving PI3K-Akt, VEGF, and cGMP-PKG pathways, while hepatotoxicity implicated VEGF, PI3K-Akt, and estrogen signaling. Core components (e.g., dihydrochelerythrine, oxysanguinarine) exhibited strong binding to targets (binding energy ≤ −5.0 kcal/mol, partial ≤ −7.0 kcal/mol). Immune infiltration analysis linked core targets to macrophages M2 and γδ T cells.

**Conclusion:**

*Chelidonii Herba* treats COPD primarily through alkaloids modulating shared targets (CASP3, PPARG, PTGS2) via PI3K-Akt pathways, while concurrently inducing hepatotoxicity through VEGF and estrogen signaling. This dual efficacy-toxicity profile necessitates cautious clinical application and experimental validation to define safe therapeutic windows.

## Introduction

Chronic Obstructive Pulmonary Disease (COPD), a typical chronic inflammatory respiratory disorder, is primarily characterized by persistent airflow limitation. Recent epidemiological statistics indicate that this disease poses a significant challenge to global public health systems, having become the third leading cause of death worldwide [1]. Furthermore, predictive models suggest that annual COPD-related fatalities may exceed 5.4 million by 2060 [2]. Despite the availability of Western medications to alleviate COPD symptoms, no definitive cure exists. Consequently, exploring the therapeutic potential of Traditional Chinese medicine (TCM) for COPD holds profound clinical significance.

**Chelidonii Herba* L.*, commonly known as greater celandine, is the dried aerial part of *Chelidonium majus L.* (Papaveraceae), officially termed *Chelidonii Herba*. Classified as bitter, cold, and toxic in nature, it primarily acts on the lung and stomach meridians. Renowned for its antispasmodic, analgesic, antitussive, and antiasthmatic effects, it is traditionally indicated for cough, asthma, pertussis, and gastric spasms [3]. Professor Guo Zhenwu, a distinguished TCM master from Liaoning Province, has extensive clinical experience in treating pulmonary diseases. He frequently employs a self-developed formula, “Sanbai Decoction,” as a core prescription tailored for COPD management. The monarch drugs in this formula are *Chelidonii Herba*, *Ginkgo Semen*, and *Cynanchi Stauntonii Rhizoma Et Radix* [4]. Modern pharmacological studies reveal that chelerythrine, a major constituent of *Chelidonii Herba*, inhibits respiratory inflammation [5], while sanguinarine exhibits antimicrobial, antifungal, and antitumor activities [6–8]. However, clinical reports highlight potential hepatotoxicity [9,10], including hemolytic anemia and acute hepatitis [11,12]. Leveraging bioinformatics techniques to elucidate the pharmacological and toxicological mechanisms of *Chelidonii Herba*, while defining its safe therapeutic window, is thus critically important for clinical practice.

Rooted in systems biology theory, network pharmacology and network toxicology focus on drug efficacy and toxicity evaluation, respectively. Network pharmacology constructs interaction networks among drugs, targets, and diseases to analyze compound-disease relationships [13], whereas network toxicology employs similar modeling approaches to predict and assess drug toxicity profiles [14]. Molecular docking calculates ligand-receptor binding free energy to predict binding affinity and biological activity [15,16]. Concurrently, emerging evidence underscores the role of immune mechanisms in COPD pathogenesis and progression [17], necessitating the evaluation of immune cell infiltration to identify novel immunotherapeutic targets. These methodologies synergize as follows: network models delineate mechanistic pathways, molecular docking validates target engagement, and immune infiltration analysis deciphers immunoregulatory mechanisms. This study integrates a multidimensional bioinformatics framework to preliminarily unveil the multi-target pharmacological network of *Chelidonii Herba* in treating COPD and its hepatotoxicity pathways. By balancing therapeutic efficacy and hepatotoxicity risks, this work aims to inform evidence-based risk-benefit assessments for clinical applications.

## Materials and methods

### Acquisition of active components and corresponding targets of *Chelidonii herba*

The term “*Chelidonii Herba*” was queried in the TCMSP database (https://old.tcmsp-e.com) to retrieve all components under the “Ingredients” column. Active components were filtered based on pharmacokinetic criteria: oral bioavailability (OB) ≥30% and drug-likeness (DL) ≥0.18. Targets corresponding to these active components were extracted from the “Related Targets” column and processed through the STRING database (https://string-db.org/) with species set to *Homo sapiens* and “Multiple proteins” mode. The 2D structures of filtered components were obtained via PubChem (https://pubchem.ncbi.nlm.nih.gov/) and uploaded to the PharmMapper database (https://www.lilab-ecust.cn/pharmmapper/submitfile.html) for additional target prediction. SMILES strings of the components were retrieved from PubChem and submitted to SwissTargetPrediction (http://www.swisstargetprediction.ch/) to identify potential targets. Duplicate targets were removed to finalize the target list.

Database retrieval dates: TCMSP (2025-05-02), SwissTargetPrediction (2025-05-02), PharmMapper (2025-05-02).

### “Drug-component-target” network analysis

Cytoscape software was used to construct a network. Active components and their targets were imported to visualize the “drug-component-target” network. Node degree values were calculated and ranked in descending order using Excel to identify the top 10 active components.

### Acquisition of COPD and hepatotoxicity targets

COPD-related targets were retrieved from GeneCards (https://www.genecards.org) and OMIM (https://omim.org/) using the search term “chronic obstructive pulmonary disease.” Hepatotoxicity targets were obtained using “Drug-Induced Liver Injury,” “Liver toxic,” and “Liver toxicity” as keywords.

Database retrieval dates: GeneCards (2025-05-03), OMIM (2025-05-03).

### Identification of overlapping targets between *Chelidonii herba* and COPD/hepatotoxicity

Venn diagrams were generated using the Weishengxin platform (http://www.bioinformatics.com.cn) [18] to intersect *Chelidonii Herba* targets with COPD targets and hepatotoxicity targets, respectively.

### Construction of protein-protein interaction (PPI) networks and core target screening

STRING database was used to analyze PPI networks of overlapping targets (COPD-related and hepatotoxicity-related) with “Multiple proteins” mode and a confidence threshold of 0.400. CytoNCA plugin in Cytoscape calculated betweenness centrality (BC), closeness centrality (CC), and degree centrality (DC) for unweighted networks. Targets exceeding median values for all parameters after iterative filtering in R were defined as core targets.

### GO and KEGG enrichment analyses

Gene Ontology (GO) enrichment analysis covering biological processes, cellular components, and molecular functions, along with KEGG pathway analysis, was performed for overlapping targets using Metascape (https://metascape.org/). The KEGG pathway enrichment specifically utilized the KEGG PATHWAY database (https://www.kegg.jp/kegg/pathway.html), which provides manually curated molecular wiring diagrams representing interaction and reaction networks within biological systems [19–21]. These pathway maps integrate four key elements: (1) molecular interactions including protein-protein and protein-compound interactions, (2) signaling cascades, (3) metabolic conversions, and (4) disease-related network variations. This analysis leveraged KEGG’s systematic integration framework for large-scale molecular datasets and employed the KEGG Orthology (KO) system for functional annotation, enabling precise mapping of gene products to these comprehensive pathway networks. Results were visualized as bubble plots for GO terms and Sankey diagrams for KEGG pathways via the Weishengxin platform.

Analysis date: Metascape (2025-05-08), KEGG PATHWAY (2025-05-08).

### Construction of “drug-component-target-pathway” network

Top 5 active components (by degree), 20 key pathways, and core targets were integrated into a network using Cytoscape to visualize their interrelationships.

### Molecular docking

Top 5 core targets (by degree) for COPD and hepatotoxicity were queried in Uniprot (https://www.uniprot.org/) to obtain PDB IDs. Protein structures were retrieved from RCSB-PDB (http://www.pdb.org/), and 2D structures of active components were downloaded from PubChem. PyMOL and AutoDock Tools preprocessed ligands and receptors, followed by docking simulations using AutoDock Vina 1.1.2. PyMOL visualized binding modes.

### GEO database processing

Gene Expression Omnibus (GEO) was queried for “chronic obstructive pulmonary disease” datasets in *Homo sapiens*. The microarray dataset GSE55962 (Affymetrix Human Genome U219 Array), containing 24 COPD and 82 healthy samples, was selected. R language corrected batch effects and normalized data for visualization.

Dataset download date: GSE55962 (2025-05-15).

### Immune infiltration analysis

The R package CIBERSORT deconvoluted immune cell profiles in GSE55962 using linear support vector regression. Immune cell proportions in COPD vs. healthy samples were compared, and correlations between core targets and immune cell subtypes were analyzed.

## Results and analysis

### Active components and targets of *Chelidonii herba*

Active components and targets of *Chelidonii Herba* were identified using TCMSP, SwissTargetPrediction (STP), and PubChem databases. Twenty active components were retrieved (Table 1). From the 20 active components in TCMSP, a preliminary set of 298 targets was retrieved. These targets were uploaded to STRING database with “Multiple proteins” and *Homo sapiens* settings. PharmMapper (PM) screening (Norm Fit > 0.8) of 20 components’ 2D structures from PubChem generated 195 targets. SwissTargetPrediction (STP) analysis of SMILES strings (Probability >0.2) identified 54 targets. After deduplication via R language, 108 unique high-confidence targets were finalized.

**Table 1:** Active components of *Chelidonii Herba.*

No.	MOL ID	Molecule Name	OB^a^ (%)	DL^b^
1	MOL000217	(S)-Scoulerine	32.28	0.54
2	MOL000729	Oxysanguinarine	46.97	0.87
3	MOL000787	Fumarine	59.26	0.83
4	MOL001454	berberine	36.86	0.78
5	MOL001455	(S)-Canadine	53.83	0.77
6	MOL001458	coptisine	30.67	0.86
7	MOL001460	Cryptopin	78.74	0.72
8	MOL001461	Dihydrochelerythrine	32.73	0.81
9	MOL001462	Dihydrochelirubine	55.29	0.86
10	MOL001463	Dihydrosanguinarine	59.31	0.86
11	MOL001465	chelamidine	31.01	0.86
12	MOL001466	(+/-)-Homochelidonine	36.84	0.84
13	MOL001467	Luteanin	55.63	0.55
14	MOL001469	methoxychelidonine	32.21	0.84
15	MOL001473	rhoeadine	63.51	0.83
16	MOL001474	sanguinarine	37.81	0.86
17	MOL001475	beta-Isosparteine	59.81	0.21
18	MOL001476	(S)-Stylopine	51.15	0.85
19	MOL001481	chelidonine	48.32	0.86
20	MOL001482	chelilutine	53.55	0.87

^a^OB:Oral bioavailability.

^b^DL:Drug-likeness.

### “Drug-component-target” network construction

**Fig 1:**
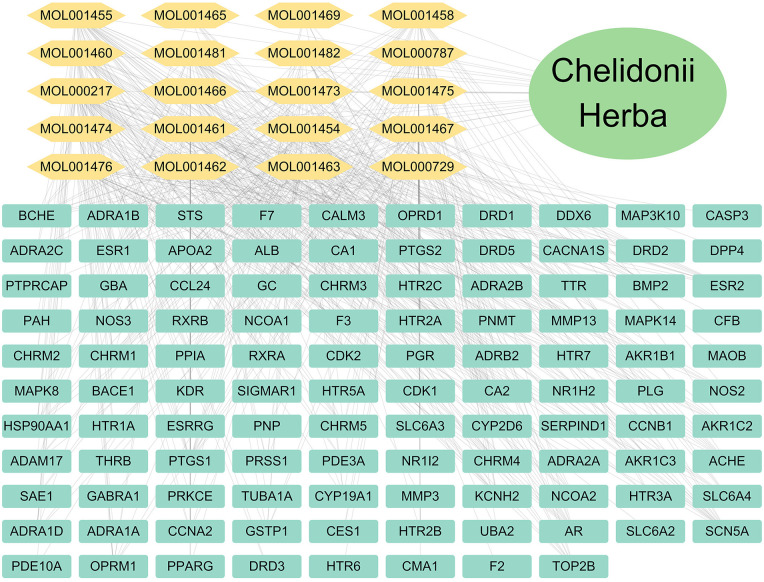
Drug-component-target network of *Chelidonii herba.*

The “drug-component-target” network (Fig 1) was visualized in Cytoscape using Mol IDs and targets. Degree values were ranked to identify the top 10 active components (Table 2): (S)-Scoulerine, (S)-Canadine, Luteanin, berberine, chelidonine, Cryptopin, Dihydrochelerythrine, (S)-Stylopine, coptisine, and Oxysanguinarine.

**Table 2:** Top 10 active components by degree value.

Category	Active Component	Structure Reference	Degree
Protoberberine-type alkaloids	(S)-Scoulerine	S1A Fig	56
	(S)-Canadine	S1B Fig	42
	Luteanin	S1C Fig	42
	berberine	S1D Fig	37
	Cryptopin	S1E Fig	32
	(S)-Stylopine	S1F Fig	31
	coptisine	S1G Fig	28
Benzophenanthridine alkaloids	chelidonine	S1H Fig	33
Dihydrobenzophenanthridine alkaloids	Dihydrochelerythrine	S1I Fig	32
	Oxysanguinarine	S1J Fig	26

Remarkably, our screening identified 20 alkaloids with OB ≥ 30% (Table 1), suggesting favorable theoretical oral bioavailability. However, it must be emphasized that actual human pharmacokinetics remain unverified. Specifically, the key component sanguinarine (SA) demonstrated rapid absorption (peak time Tmax = 1.75 h) in rats following oral gavage, while human liver microsome studies confirm its extensive metabolism via reduction to dihydrosanguinarine (DHSA) mediated by CYP1A1 and CYP1A2 enzymes. In contrast, chelerythrine (CHE) achieved a maximum plasma concentration (Cmax) of only 5.06 ng/mL with rapid elimination (half-life T1/2 = 2.82 h) after oral administration in rats. Furthermore, CHE is primarily metabolized to dihydrochelerythrine (DHCHE) in vivo [22].

### COPD and hepatotoxicity target screening

GeneCards and OMIM databases were queried for “chronic obstructive pulmonary disease” (Relevance score >10), “Drug-Induced Liver Injury,” “Liver toxic,” and “Liver toxicity.” After deduplication, 5,158 COPD-related targets and 4,930 hepatotoxicity-related targets were identified.

### Overlapping targets between drug components and diseases

**Fig 2:**
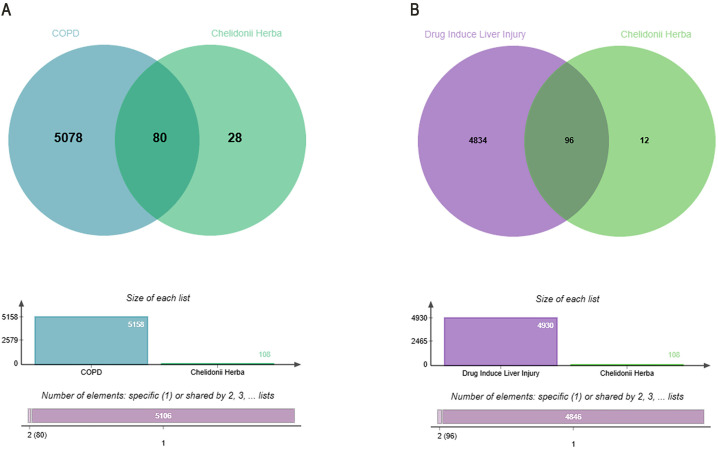
Venn diagrams of shared targets between *Chelidonii herba* and diseases. (A) COPD; (B) Hepatotoxicity.

Venn diagrams (Fig 2A and 2B) visualized overlapping targets between *Chelidonii Herba* and COPD/hepatotoxicity. “*Chelidonii herba*-COPD” shared 80 targets (Fig 2A), while “*Chelidonii herba*-hepatotoxicity” shared 96 targets (Fig 2B).

### PPI network construction and core target identification

**Fig 3:**
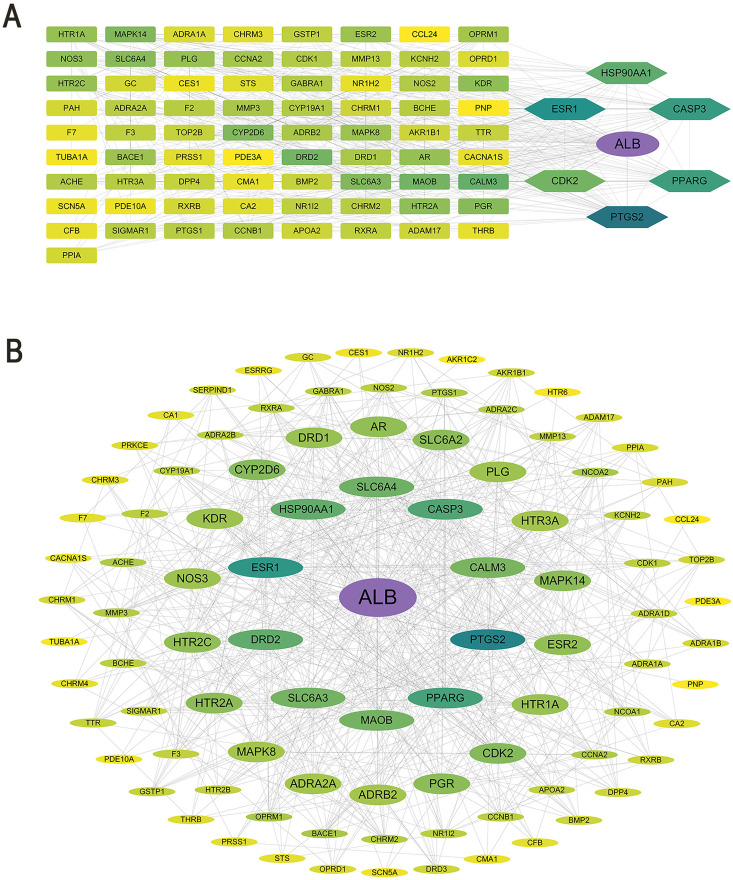
Protein-protein interaction networks of shared targets. (A) *Chelidonii herba*-COPD targets; (B) *Chelidonii herba*-hepatotoxicity targets.

STRING database analyzed PPI networks of overlapping targets (COPD: 80 nodes, 492 edges; hepatotoxicity: 96 nodes, 619 edges) with “Medium confidence (0.400)” threshold. CytoNCA calculated BC, CC, and DC for unweighted networks. Iterative R filtering retained targets exceeding median values for all parameters, yielding 7 core targets for both COPD (Fig 3A) and hepatotoxicity (Fig 3B).

### GO enrichment analysis

**Fig 4:**
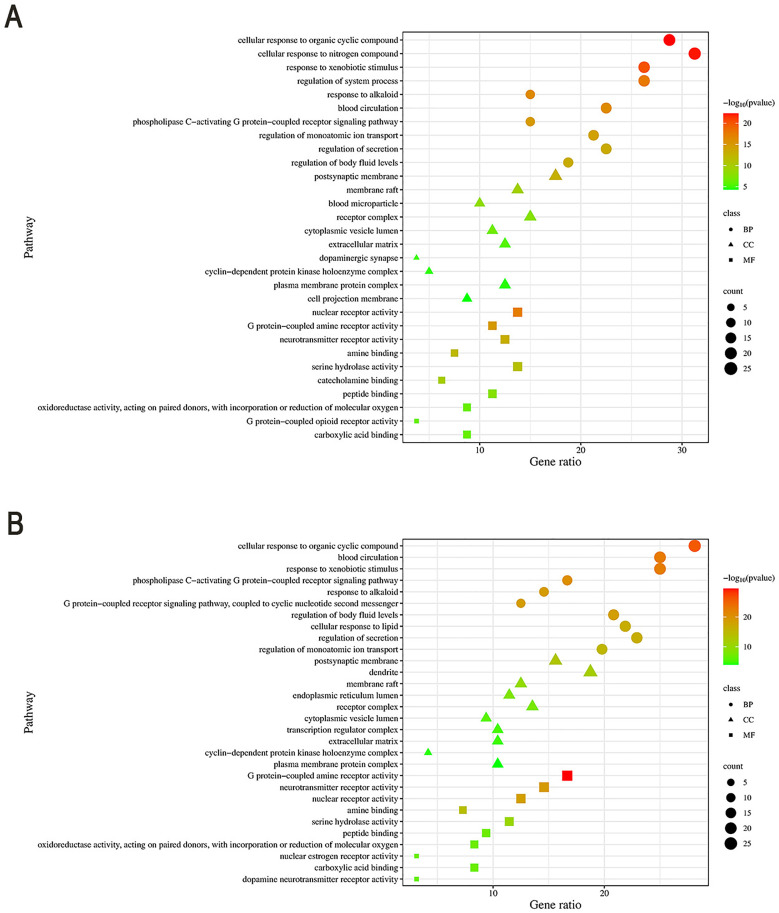
Gene Ontology enrichment analysis. (A) *Chelidonii herba*-COPD targets; (B) *Chelidonii herba*-hepatotoxicity targets.

Metascape performed GO analysis (*P < 0.01,FDR < 0.01* [23]). “*Chelidonii herba*-COPD” yielded 4,292 terms (3,328 BP, 342 CC, 622 MF); “***Chelidonii herba***-hepatotoxicity” yielded 4,465 terms (3,431 BP, 344 CC, 690 MF). Top 10 terms per category (sorted by P-value) were visualized as bubble plots (Fig 4A and 4B).

### KEGG enrichment analysis

**Fig 5:**
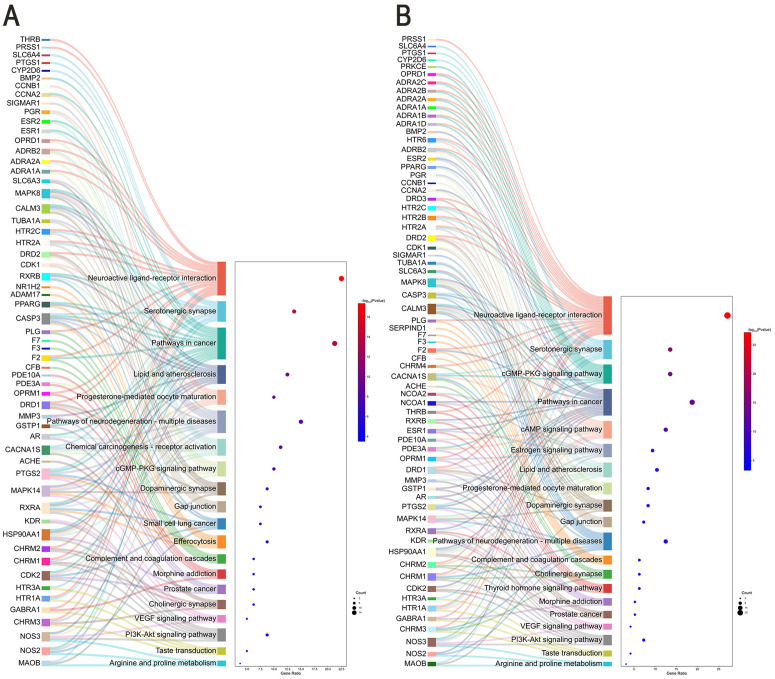
KEGG pathway enrichment analysis. (A) *Chelidonii herba*-COPD targets; (B) *Chelidonii herba*-hepatotoxicity targets. Pathway analysis based on data from the KEGG database [18].

KEGG analysis (*P < 0.01,FDR < 0.01*) identified 216 pathways for “***Chelidonii herba***-COPD” and 217 for “***Chelidonii herba***-hepatotoxicity.” Top 20 pathways (sorted by P-value) were visualized as Sankey diagrams (Fig 5A and 5B).

### “Drug-component-core target-pathway” network

**Fig 6:**
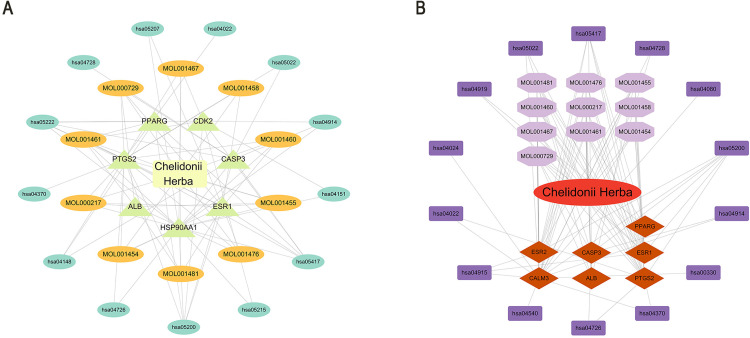
Integrated drug-component-target-pathway networks. (A) COPD treatment; (B) Hepatotoxicity.

Cytoscape visualized networks integrating top 10 components, 7 core targets, and 20 KEGG pathways for COPD treatment (Fig 6A) and hepatotoxicity (Fig 6B).

### Molecular docking results

**Fig 7:**
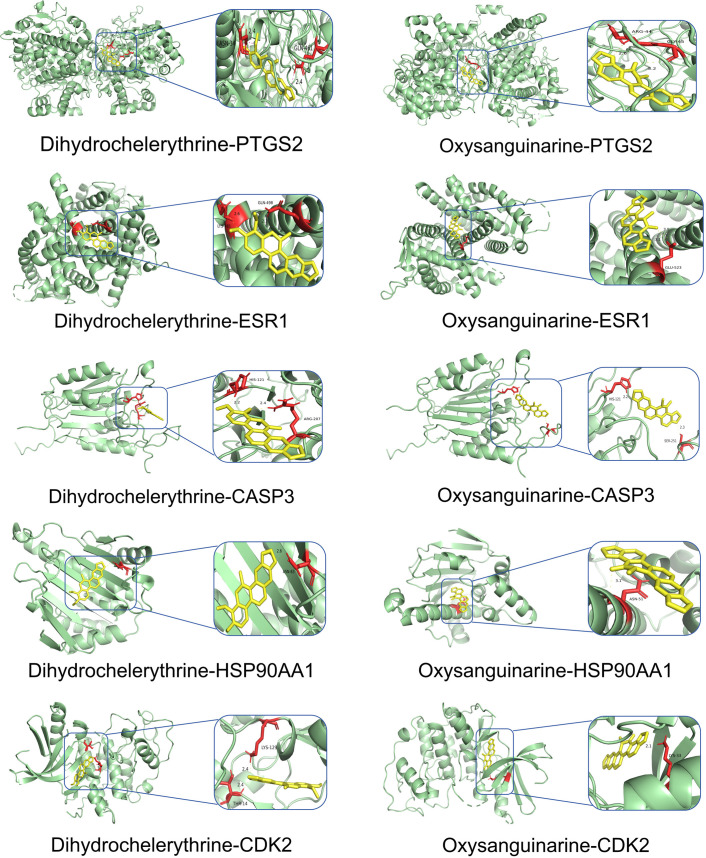
Molecular docking visualization: Dihydrochelerythrine and oxysanguinarine with core targets.

**Fig 8:**
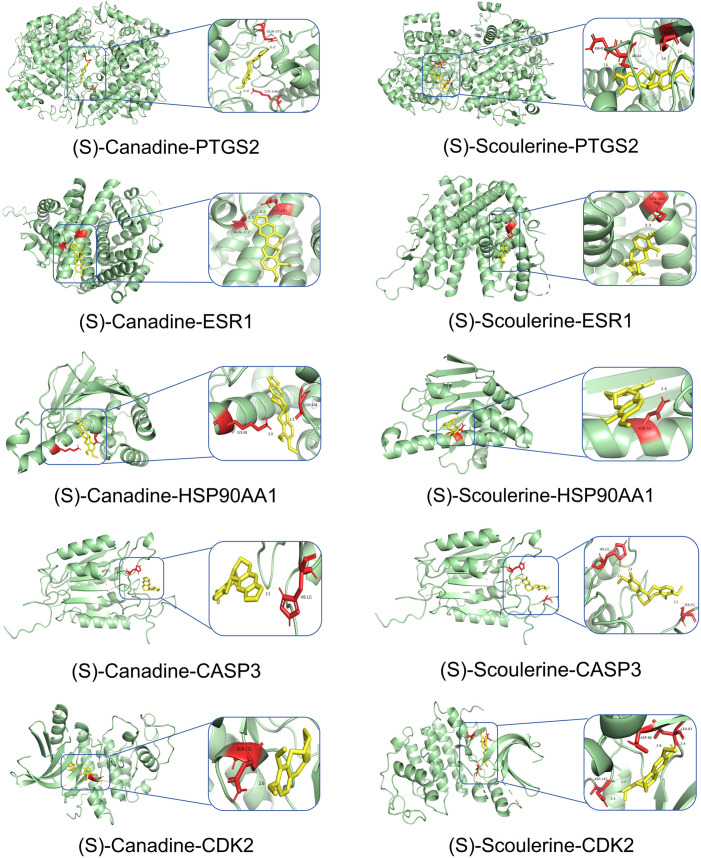
Molecular docking visualization: (S)-Canadine and (S)-Scoulerine with core targets.

**Fig 9:**
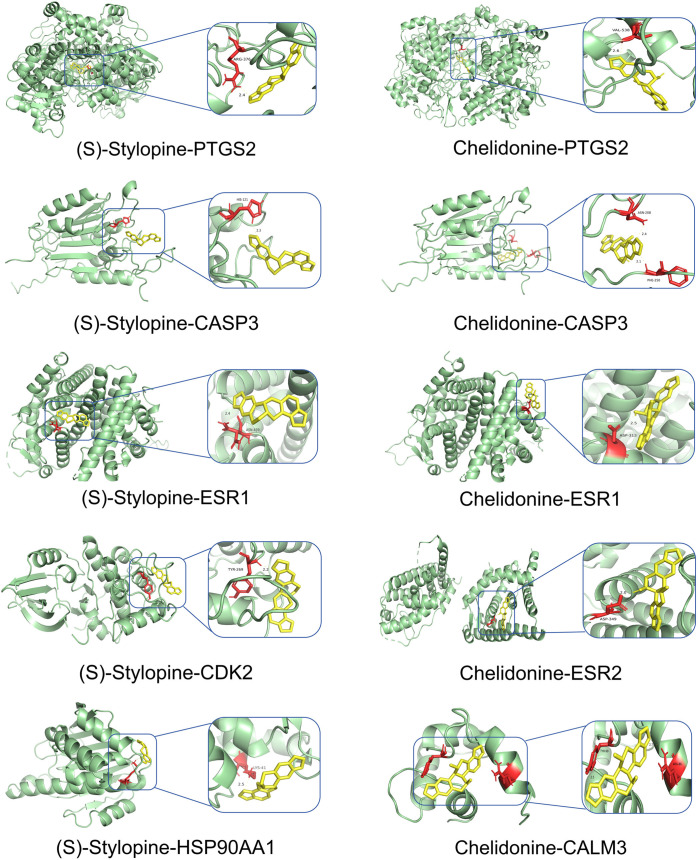
Molecular docking visualization: (S)-Stylopine and chelidonine with core targets.

**Fig 10:**
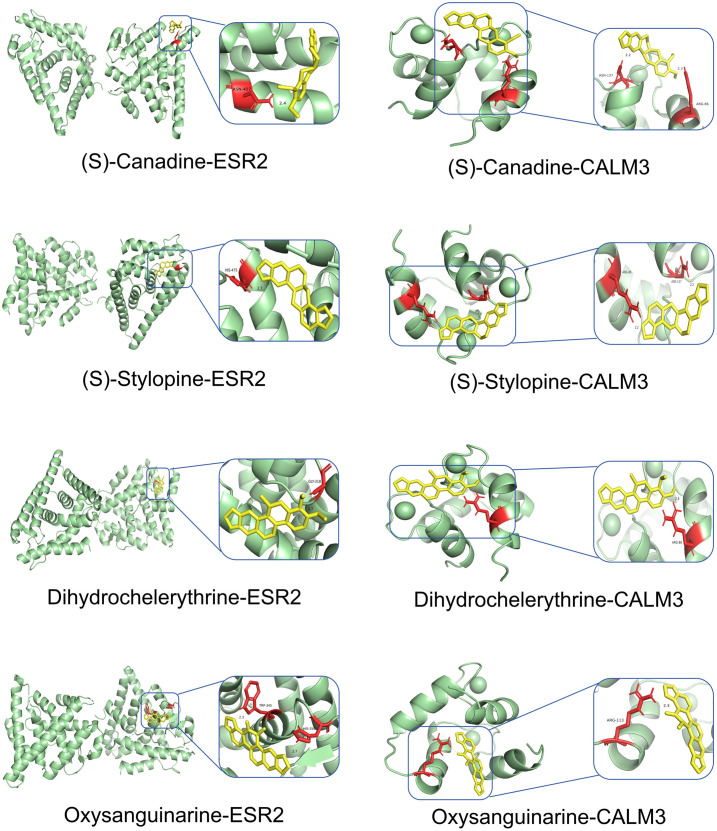
Molecular docking visualization: Dihydrochelerythrine, oxysanguinarine, (S)-Stylopine, and (S)-Canadine with core targets.

**Fig 11:**
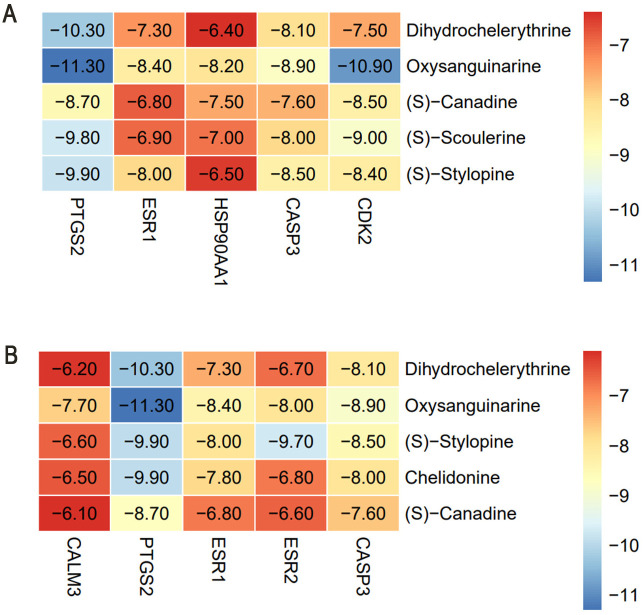
Heatmaps of molecular docking binding energies. (A) *Chelidonii herba*-COPD; (B) *Chelidonii herba*-hepatotoxicity.

Network Analyzer calculated network topology parameters. For each ligand-target complex, molecular docking simulations were performed with 20 independent runs. Representative binding poses with optimal binding energies are visualized in Figs 7–10. The mean binding affinity across all runs was below −5.0 kcal/mol for all 38 complexes, consistent with thresholds commonly used in molecular docking literature [24–26] where values < −5.0 kcal/mol suggest significant binding activity. Statistical analysis revealed narrow 95% confidence intervals (mean width: 0.53 kcal/mol), with all upper bounds remaining below −5.0 kcal/mol, further confirming binding stability. Thirty-two complexes (84.2%) exhibited strong binding affinities (mean affinity ≤ −6.0 kcal/mol), including 5 with exceptional stability (<−8.0 kcal/mol): Oxysanguinarine-CDK2 (−9.17 kcal/mol), Chelidonine-PTGS2 (−9.085 kcal/mol), and (S)-Stylopine-PTGS2 (−9.005 kcal/mol). Fig 11A and 11B display heatmaps of the optimal binding energies for each complex.

### Immune infiltration analysis

**Fig 12:**
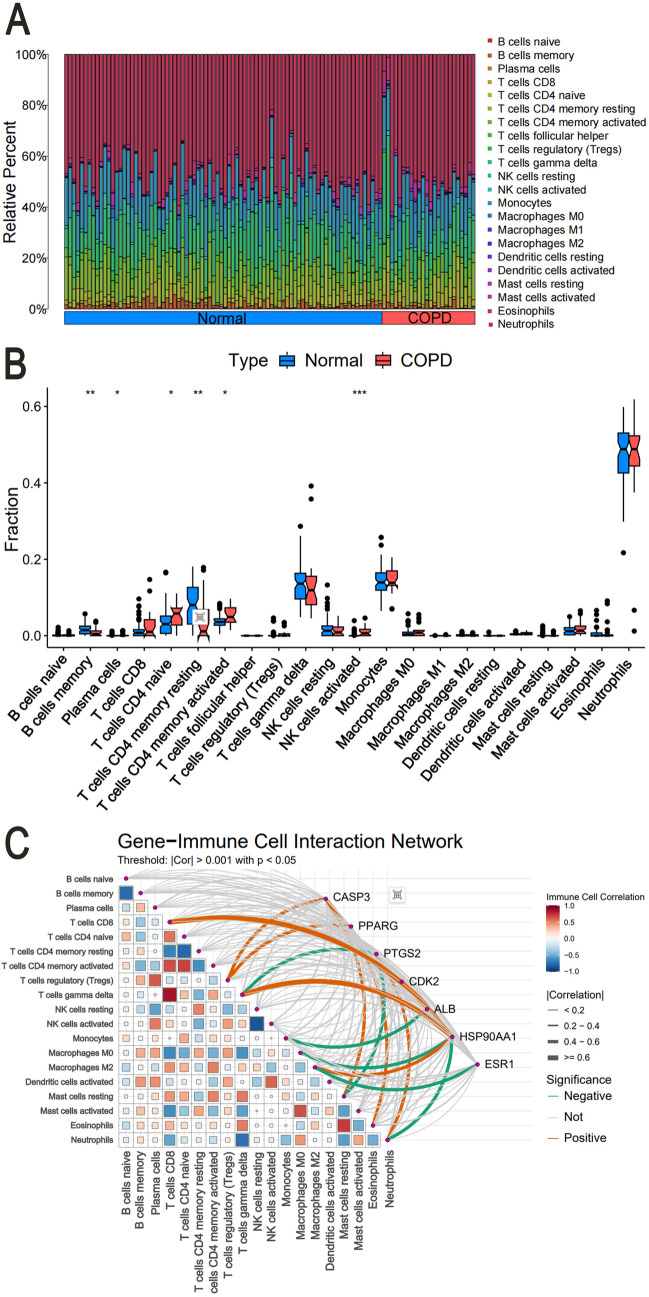
Immune profiling analysis of COPD. (A) Immune cell proportions across samples; (B) Differential immune cell abundance; (C) Core gene-immune cell correlations.

CIBERSORT analyzed immune cell proportions in GSE55962. Bar plots (Fig 12A) showed 22 immune cell types per sample. Box plots (Fig 12B) revealed that in COPD patients, activated NK cells were relatively higher, whereas in healthy controls, memory B cells and resting CD4 + memory T cells showed relatively higher abundance. Correlation analysis (Fig 12C) linked core target gene expression to immune cell infiltration levels.

## Discussion

### Pharmacodynamic material basis of *Chelidonii herba* in COPD treatment

The primary active components of *Chelidonii Herba* are alkaloids, including chelidonine, chelerythrine, berberine, coptisine, sanguinarine [27]. Chelidonine, a benzophenanthridine alkaloid, exhibits spasmolytic and relaxant effects, effectively inhibiting spasms in bronchial, gastrointestinal, and urinary smooth muscles, along with antibacterial activity against Mycobacterium tuberculosis (in vivo) and Gram-positive bacteria (e.g., Streptococcus pyogenes, Streptococcus pneumoniae) in vitro [28]. chelerythrine, a quaternary benzophenanthridine alkaloid, attenuates LPS-induced acute lung injury by inhibiting NF-κB activation and Nrf2 nuclear translocation, reducing proinflammatory cytokines (TNF-α, IL-6, IL-1β), and alleviating pulmonary edema and neutrophil infiltration. It also disrupts bacterial membranes, induces protein leakage, and blocks protein synthesis in Gram-positive bacteria (e.g., Staphylococcus aureus, MRSA, ESBLs) [29].

Berberine demonstrates dose-dependent anti-inflammatory effects in COPD rat models by suppressing airway inflammation [30]. Coptisine, found in Ranunculaceae and Papaveraceae plants, exhibits antibacterial, anticancer, and glycemic/lipid-regulating activities [31]. Sanguinarine additionally shows antioxidant, anti-inflammatory, and antitumor properties [32]. Isocorydine enhances immunity, improves microcirculation, and inhibits bacterial growth [33]. Total alkaloids of *Chelidonii Herba* reduce cough frequency and enhance expectoration [34]. Modern medicine recognizes that specific inflammation underlies the entire course of COPD. Among the 20 active components screened in this study, the aforementioned five alkaloids have been confirmed to possess anti-inflammatory and antibacterial activities. These components treat COPD by acting on multiple targets to modulate relevant pathways.

### Mechanistic analysis of *Chelidonii herba* in COPD treatment

Seven core targets were identified from the PPI network: *CASP3*, *PPARG*, *PTGS2*, *CDK2*, *ALB*, *HSP90AA1*, and *ESR1*. *CASP3* (Caspase-3) mediates apoptosis, tissue differentiation, and neurodevelopment [35]. *PPARG* activation counteracts smoke-induced inflammation by inhibiting proinflammatory chemokines [36]. *PTGS*2 upregulation correlates with COPD pathogenesis [37]. As a key regulator of the cell cycle, *CDK2* triggers cough by mediating inflammatory cell proliferation and mediator release, which activates airway neural sensitivity signals [38]. *ALB* (albumin) modulates osmotic pressure, drug transport, and neutrophil-endothelial interactions to mitigate lung injury [39,40]. Low serum albumin (<40 g/L) doubles respiratory mortality [41]. *HSP90AA1*, a member of the heat shock protein family, shows altered expression in COPD patients and modulates TNF secretion in monocytes to participate in local inflammatory responses [42]. Multiple subtypes of estrogen receptors (*ES*R) are widely distributed in lung tissue. Altered mRNA expression of estrogen-metabolizing enzymes in COPD lungs suggests estrogen involvement in the disease [43].

KEGG analysis highlighted pathways like PI3K-Akt, VEGF, and cGMP-PKG. The PI3K-Akt pathway regulates inflammatory mediator release, immune cell activation, and antioxidant responses via AKT phosphorylation, thereby contributing to airway remodeling [44,45]. VEGF signaling links to pulmonary vascular proliferation; reduced VEGF in COPD sputum correlates with alveolar damage [46,47]. The VEGF signaling pathway plays a central role in pulmonary vascular remodeling in COPD by activating the PI3K-Akt pathway, driving endothelial cell proliferation and migration. Experimental evidence confirms that VEGF overexpression in COPD lung tissue correlates with alveolar destruction and arterial thickening [48]. Notably, chronic hypoxia induces VEGF transcription via HIF-1α, leading to a pathological cascade that promotes vascular proliferation in COPD [49]. VEGF and b-FGF are both involved in airway remodeling through mechanisms such as vascular regeneration and mast cell proliferation, respectively [50]. These mechanisms align with our prediction that *Chelidonii Herba* alkaloids (e.g., dihydrochelerythrine) modulate the VEGF/PI3K-Akt axis—paralleling interventions by TCM formulas targeting this pathway. The cGMP-PKG signaling pathway plays vital roles in multiple biological processes, including anti-atherosclerosis, anti-hypertension, and modulation of smooth muscle relaxation and contraction [51].

Molecular docking confirmed strong binding between the active components (Dihydrochelerythrine, Oxysanguinarine, (S)-Canadine, (S)-Scoulerine, and (S)-Stylopine) and core targets (PTGS2, ESR1, HSP90AA1, CASP3, CDK2), suggesting that *Chelidonii Herba* may treat COPD patients through multi-target and multi-pathway mechanisms. Immune infiltration analysis linked core targets to macrophages M2, γδ T cells, and mast cells, warranting further immunomodulatory studies.

The PI3K-Akt pathway regulates autophagy and oxidative stress in COPD. It upregulates Nrf2 to promote antioxidant protein expression, thereby alleviating oxidative stress and reducing lung injury. Regarding autophagy regulation, under pathological conditions such as nutrient deficiency or hypoxia, the pathway is inhibited, leading to autophagy activation [52]. Furthermore, inhibition of the PI3K-Akt pathway reduces TGF-β1 expression, thereby alleviating the airway remodeling process in COPD [53]. Integrated analysis predicts that *Chelidonii Herba* alkaloids (e.g., dihydrochelerythrine) may restore autophagy-oxidative balance by modulating PI3K-Akt signaling.

However, it must be emphasized that these bioinformatics-based predictions urgently require rigorous validation through a multidimensional experimental approach. At the in vivo level, the cigarette smoke (CS) combined with lipopolysaccharide (LPS)-induced COPD animal model, which has been proven to effectively mimic the multifactorial pathogenic features of chronic airway inflammation and lung parenchymal injury [54], is recommended. At the in vitro level, models utilizing CSE/LPS stimulation in alveolar epithelial cells (e.g., A549; 10% CSE + 1000 ng/mL LPS) and macrophages (e.g., RAW264.7; 5% CSE + 75 ng/mL LPS) for 48 hours [55] are suggested. These models should focus on validating the modulatory effects of key *Chelidonii Herba* alkaloids (e.g., dihydrochelerythrine, oxysanguinarine) on inflammatory pathways, oxidative stress responses, and the activity of core targets with validated binding affinity (e.g., CASP3, PTGS2). The synergistic application of these in vivo and in vitro models will be crucial to decode their functional mechanisms in COPD pathogenesis and definitively elucidate the material basis underlying the lung-protective efficacy.

### Toxicological material basis of *Chelidonii herba*-induced hepatotoxicity

Alkaloids (chelidonine, chelerythrine, coptisine, sanguinarine, protopine) are primary hepatotoxicants in *Chelidonii Herba*. The metabolic clearance of chelidonine primarily relies on CYP isozymes (3A4/1A2/2C19/2D6)-mediated oxidation, generating demethylated metabolites. The phenolic hydroxyl groups in these metabolites undergo oxidation to form quinoid compounds, which conjugate with glutathione to yield quinone-thioethers, causing hepatic glutathione depletion [9,10,56]. Coptisine induces cytotoxicity in human HL-7702 hepatocytes and rat hepatocytes [57]. Sanguinarine and protopine also exhibit hepatotoxicity, requiring toxicological validation.

### Mechanistic analysis of *Chelidonii herba*-induced hepatotoxicity

Seven core targets (*PPARG*, *ESR1*, *CASP3*, *PTGS2*, *ESR2*, *CALM3*, *ALB*) and pathways (VEGF, PI3K-Akt, cGMP-PKG, cAMP, Estrogen signaling) were implicated. As a member of the PPAR family, *PPARG* regulates genes involved in glucose/lipid metabolism and inflammatory responses. Activation of the PPAR family in hepatocytes enhances oxidative stress, triggers cell proliferation and death, and may ultimately promote hepatocellular carcinogenesis [58]. *ESR1*, a protein regulating cell proliferation and inflammatory responses, when impaired, abolishes estrogen-mediated inhibition of hepatocyte proliferation, leading to structural damage and functional disruption of liver cells [59]. Research has confirmed that *CASP3* serves as a biomarker for apoptosis in liver cells [60]. PTGS2 is closely associated with inflammatory responses, and excessive inflammation leads to liver injury [61]. Recent studies confirm that pathological neovascularization permeates all stages of hepatic inflammation, injury, and fibrotic repair [62]. The VEGF signaling pathway mediates inflammation-related angiogenesis [63], wherein the hypoxia-inducible HIF-1 pathway transcriptionally activates VEGF signaling to promote angiogenesis under hypoxic conditions [64], while specific cytokines stimulate VEGF-mediated angiogenesis through the RAP1 signaling pathway [65]. Concurrently, as a downstream effector of VEGF, the PI3K-Akt signaling pathway plays critical roles in angiogenesis and apoptosis during liver injury [66].

Moreover,hepatic fibrosis involves a multifactorial pathological process characterized by hepatic stellate cell (HSC) activation, inflammatory responses, and extracellular matrix (ECM) deposition. Crucially, activation of the PI3K/Akt pathway modulates HSC proliferation and migration, thereby facilitating the development of hepatic fibrosis [67]. Beyond its role in maintaining female secondary sexual characteristics, the estrogen signaling pathway causes hepatorenal toxicity by regulating specific genes [68].

Molecular docking revealed strong binding between the active components (Dihydrochelerythrine, Oxysanguinarine, (S)-Stylopine, chelidonine, and (S)-Canadine) and core targets (CALM3, PTGS2, ESR1, ESR2, CASP3), suggesting that *Chelidonii Herba* may induce hepatotoxicity through multi-target mechanisms.

Collectively, these findings suggest VEGF, PI3K-Akt, and estrogen signaling may represent common pathways underlying *Chelidonii Herba* hepatotoxicity. To advance experimental validation of such multi-pathway hepatotoxicity, established methodologies could be considered as reference approaches; for instance, in vitro systems like rat primary hepatocytes may assess transporter inhibition (e.g., Oatp1b2) [69], while 3D hepatosphere models combined with spatially-resolved metabolomics (e.g., MALDI-MSI) could monitor spatiotemporal changes in metabolites closely related to cell viability [70]. Complementary in vivo models such as acetaminophen (APAP)-induced hepatic necrosis provide options for direct toxicity evaluation, and rifampicin-induced cholestasis models may probe excretory dysfunction [71]. These methodological examples illustrate potential avenues to investigate dose-toxicity relationships and species-specific risks in future studies.

It is crucial to acknowledge that while this study focused on the monotherapeutic mechanisms of *Chelidonii Herba*, clinical applications typically involve formula compatibility. Existing clinical research demonstrates that TCM formulas can achieve “efficacy enhancement and toxicity reduction” (Zeng Xiao Jian Du) through synergistic interactions among components [72]. For instance, *Tripterygium wilfordi Hook.f.* combined with *Paeoniae Radix Alba* alleviates its hepatotoxic oxidative stress; *Scolopendra* extract paired with Ganoderma and Ginseng Radix Et Rhizoma mitigates liver injury via anti-lipid peroxidation. Formula components can simultaneously regulate multiple pathways for comprehensive intervention, such as Yinchen Sini Decoction (Yīnchén Sìnì Tāng) inhibiting inflammatory pathways while reducing intrahepatic bile acid stasis, Yigan Capsule (Yìgān Jiāonáng) co-regulating JAK2/STAT3 and SOCS-3 to suppress inflammation, or Chaihu Shugan San (Chàihú Shūgān Sǎn) integrating anti-inflammatory and antioxidant pathways to ameliorate drug-induced liver injury [73].

### Clinical safety implications and future perspectives

*Chelidonii Herba* dosage is recorded as 9–18 g in the Chinese Pharmacopoeia (ChP) and 1–3 Qian (≈3–9 g) in the Chinese Materia Medica. This study confirms that its alkaloids mediate both COPD efficacy and hepatotoxicity, exhibiting significant overlap in chemical structures, targets (e.g., CASP3, PPARG), and signaling pathways (PI3K-Akt, VEGF, cGMP-PKG). A critical risk-benefit assessment must weigh its potent anti-inflammatory effects against hepatotoxicity risks. In this context, inter-individual metabolic variability and formula compatibility strategies may critically influence the therapeutic window. Clinical application may require heightened vigilance in specific populations: contraindications may include patients with pre-existing hepatic impairment due to potential glutathione depletion, those taking CYP3A4/2C19-metabolized drugs (e.g., warfarin) given metabolic competition risks, and pregnant women based on estrogen pathway modulation. This intrinsic “efficacy-toxicity duality” complicates hepatotoxicity avoidance within traditional dosage ranges. Critically, the ChP defines dosage (9–18 g) but lacks safety thresholds for toxic alkaloids and evidence-based toxicity grading (e.g., using LD50). Variability in metabolism and extraction further obscures toxicity thresholds. While frameworks like Lou’s classification [74] offer qualitative assessment, quantitative data (e.g., LD50, hepatic accumulation kinetics) are absent. Establishing the alkaloids’ toxic dose window relative to the ChP range is thus imperative to inform safety standards. Until then, clinical use requires: starting with minimal doses, gradual titration, individualized adjustment, and strict avoidance of long-term/excessive use. Although hepatotoxicity risk exists within the ChP range, a controllable therapeutic window is likely. Future work must define hepatic accumulation kinetics and dose-response relationships via pharmacokinetics, alongside process optimization and compatibility strategies to simultaneously reduce toxicity and enhance efficacy.

It is important to note that these findings should be interpreted in the context of methodological constraints inherent to predictive bioinformatics approaches. The core limitation of this study lies in its predictive nature where despite rigorous methodologies all findings fundamentally depend on public database coverage and algorithmic accuracy lacking experimental validation. Crucially the predicted interactions between core targets such as CASP3 PTGS2 and active components including dihydrochelerythrine and oxysanguinarine along with the functional roles of key pathways like PI3K-Akt and VEGF in mediating both efficacy and toxicity require urgent confirmation through molecular techniques such as Western blotting reporter gene assays and targeted gene manipulation complemented by animal model studies. Furthermore uncharacterized dose-response relationships prevent assessment of differential target regulation at varying doses which is essential for defining clinical safety windows while the study’s inability to model complex formula interactions particularly the potential synergies or antagonisms within clinically used preparations like Sanbai Decoction significantly limits translational relevance [75–77]. Additionally clinical heterogeneity in the GSE55962 dataset which may lack comprehensive stratification of COPD phenotypes could compromise the generalizability of immune infiltration results. Technical constraints further compound these issues as molecular docking outcomes vary across algorithms and large molecular weight ligands could potentially yield false positives whereas database parameters such as oral bioavailability thresholds often misrepresent true in vivo behavior exemplified by berberine’s pharmacokinetic profile. Nevertheless while these limitations necessitate caution our integrated analysis provides prioritized testable hypotheses for future validation using the proposed experimental models including COPD animal systems and hepatotoxicity assays to resolve these critical gaps.

## Conclusion

This study integrates network pharmacology, molecular docking, and immune profiling to elucidate the dual efficacy-toxicity mechanisms of *Chelidonii Herba* in COPD treatment and hepatotoxicity. We identified core targets (CASP3, PPARG, PTGS2) and pathways (PI3K-Akt, VEGF) as key mediators of both therapeutic and toxic effects, providing a mechanistic basis for its clinical hepatotoxicity risks alongside anti-inflammatory benefits. Immune infiltration further linked these targets to macrophages M2 and γδ T cells, suggesting potential immunomodulatory roles in COPD.

However, findings require validation beyond bioinformatics predictions. Limitations include dependency on database coverage, lack of experimental confirmation for target interactions, uncharacterized dose-response relationships, and insufficient modeling of formula compatibility effects. Future studies should prioritize experimental validation in COPD and hepatotoxicity models, define alkaloid toxicity thresholds through pharmacokinetics, and explore formula strategies (e.g., Sanbai Decoction) to mitigate hepatotoxicity while preserving efficacy.

### Abbreviations

**Table:** 

Abbreviation	Full term	First use section
ALB	Albumin	Results
BC	Betweenness Centrality	Methods
BP	Biological Processes (Gene Ontology)	Results
CALM3	Calmodulin 3	Results
CC	Cellular Components (Gene Ontology)/ Closeness Centrality	Methods/ Results
CDK2	Cyclin-Dependent Kinase 2	Results
cAMP	cyclic Adenosine Monophosphate	Discussion
COPD	Chronic Obstructive Pulmonary Disease	Abstract
CYP	Cytochrome P450	Discussion
DC	Degree Centrality	Methods
DL	Drug-Likeness	Table 1 footnote
ESBLs	Extended-Spectrum Beta-Lactamases	Discussion
ESR1	Estrogen Receptor 1	Results
ESR2	Estrogen Receptor 2	Results
FDR	False Discovery Rate	Methods
GEO	Gene Expression Omnibus	Methods
GO	Gene Ontology	Methods
HSP90AA1	Heat Shock Protein 90 Alpha Family Class A Member 1	Results
IL-1β	Interleukin-1 Beta	Discussion
IL-6	Interleukin-6	Discussion
KEGG	Kyoto Encyclopedia of Genes and Genomes	Methods
LPS	Lipopolysaccharide	Discussion
MF	Molecular Functions (Gene Ontology)	Results
MOL ID	Molecule Identifier (database-specific)	Table 1 header
MRSA	Methicillin-Resistant Staphylococcus aureus	Discussion
NF-κB	Nuclear Factor Kappa-Light-Chain-Enhancer of Activated B Cells	Discussion
NK cells	Natural Killer cells	Results
Nrf2	Nuclear Factor Erythroid 2–Related Factor 2	Discussion
OB	Oral Bioavailability	Table 1 footnote
OMIM	Online Mendelian Inheritance in Man	Methods
PI3K-Akt	Phosphatidylinositol 3-Kinase-Protein Kinase B	Results
PM	PharmMapper Server	Results
PPARG	Peroxisome Proliferator-Activated Receptor Gamma	Results
PPI	Protein-Protein Interaction	Methods
PTGS2	Prostaglandin-Endoperoxide Synthase 2	Results
STP	SwissTargetPrediction	Abstract
TCM	Traditional Chinese Medicine	Introduction
TCMSP	Traditional Chinese Medicine Systems Pharmacology Database and Analysis Platform	Methods
TNF-α	Tumor Necrosis Factor Alpha	Discussion
VEGF	Vascular Endothelial Growth Factor	Results

## Supporting information

S1 Fig 2D structures of active components from *Chelidonii Herba.* Chemical structures were sourced from the PubChem database (https://pubchem.ncbi.nlm.nih.gov/). Panel designations correspond to Table 2 entries: (A) (S)-Scoulerine; (B) (S)-Canadine; (C) Luteanin; (D) berberine; (E) Cryptopin; (F) (S)-Stylopine; (G) coptisine; (H) chelidonine; (I) Dihydrochelerythrine; (J) Oxysanguinarine.
(TIF)

S1 Data Chelidonii_Herba_Targets. (ZIP)

S2 Data Drug_Component_Target_Network (ZIP)

S3 Data Disease_Targets. (ZIP)

S4 Data Intersection_Targets. (ZIP)

S5 Data Core_Targets. (ZIP)

S6 Data Enrichment_Analysis. (ZIP)

S7 Data Drug_Component_CoreTarget_Pathway. (ZIP)

S8 Data Molecular_Docking. (ZIP)

S9 Data Immune_Infiltration. (ZIP)

## References

[1] Safiri S, Carson-Chahhoud K, Noori M, Nejadghaderi SA, Sullman MJM, Ahmadian Heris J, et al. Burden of chronic obstructive pulmonary disease and its attributable risk factors in 204 countries and territories, 1990-2019: results from the Global Burden of Disease Study 2019. BMJ. 2022;378:e069679. doi: 10.1136/bmj-2021-069679 35896191 PMC9326843

[2] GBD 2013 Mortality and Causes of Death Collaborators. Global, regional, and national age-sex specific all-cause and cause-specific mortality for 240 causes of death, 1990-2013: a systematic analysis for the Global Burden of Disease Study 2013. Lancet. 2015;385(9963):117–71. doi: 10.1016/S0140-6736(14)61682-2 25530442 PMC4340604

[3] Chinese Pharmacopoeia Commission. Pharmacopoeia of the People’s Republic of China: Volume I. 1st ed. Beijing: China Medical Science Press; 2020. p. 122–3. Chinese.

[4] Pang WW, Li DL, Cui YH, Yu XF, Guo ZW, Pang M, et al. Structural network algorithm method for investigating therapeutic rules of Zhenwu Guo for treating chronic obstructive pulmonary disease. Chinese J Control Endemic Dis. 2022;37(4):271–4. Chinese.

[5] Kim S-H, Hong J-H, Lee Y-C. Chelidonine, a principal isoquinoline alkaloid of Chelidonium majus, attenuates eosinophilic airway inflammation by suppressing IL-4 and eotaxin-2 expression in asthmatic mice. Pharmacol Rep. 2015;67(6):1168–77. doi: 10.1016/j.pharep.2015.04.013 26481537

[6] Gibbons S, Leimkugel J, Oluwatuyi M, Heinrich M. Activity of Zanthoxylum clava-herculis extracts against multi-drug resistant methicillin-resistant Staphylococcus aureus (mdr-MRSA). Phytother Res. 2003;17(3):274–5. doi: 10.1002/ptr.1112 12672160

[7] Wang M, Ma B, Ni Y, Xue X, Li M, Meng J, et al. Restoration of the Antibiotic Susceptibility of Methicillin-Resistant Staphylococcus aureus and Extended-Spectrum β-Lactamases Escherichia coli Through Combination with Chelerythrine. Microb Drug Resist. 2021;27(3):337–41. doi: 10.1089/mdr.2020.0044 32721267

[8] Wang P, Zheng S-Y, Jiang R-L, Wu H-D, Li Y-A, Lu J-L, et al. Necroptosis signaling and mitochondrial dysfunction cross-talking facilitate cell death mediated by chelerythrine in glioma. Free Radic Biol Med. 2023;202:76–96. doi: 10.1016/j.freeradbiomed.2023.03.021 36997101

[9] Gao L, Schmitz H-J, Merz K-H, Schrenk D. Characterization of the cytotoxicity of selected Chelidonium alkaloids in rat hepatocytes. Toxicol Lett. 2019;311:91–7. doi: 10.1016/j.toxlet.2019.04.031 31054355

[10] Zhang YY, Dong WH, Xu JJ, Zhu H, Li LD, Sun L. Identification of metabolites of chelidonine in human liver microsomes. J Shenyang Pharm Univ. 2018;35(6):477–83. Chinese. doi: 10.14066/j.cnki.cn21-1349/r.2018.06.008

[11] Han FM, Peng ZH, Song W, Zhang HM, Zhu MM, Chen Y. Identification of dauricine and its metabolites in rat urine by liquid chromatography-tandem mass spectrometry. J Chromatogr B Analyt Technol Biomed Life Sci. 2007;854(1–2):1–7. doi: 10.1016/j.jchromb.2007.03.036 17448738

[12] Benninger J, Schneider HT, Schuppan D, Kirchner T, Hahn EG. Acute hepatitis induced by greater celandine (Chelidonium majus). Gastroenterology. 1999;117(5):1234–7. doi: 10.1016/s0016-5085(99)70410-5 10535888

[13] Chen YH, Liu CX, He T, Yuan FL, Wang WX, Tian Y, et al. Network pharmacology study on Danshen decoction in treatment of diabetic cardiomyopathy. Chinese Tradition Herbal Drug. 2019;50(5):1164–74. Chinese.

[14] Yang X, Liu CX, Yuan FL, Wang WX, He T, Han S, et al. Mechanism of renal injury in rats induced by Phytolaccae Radix based on network toxicology. Chinese Tradition Herb Drug. 2019;50(20):4974–84. Chinese.

[15] Zeng Z, Hu J, Xiao G, Liu Y, Jia D, Wu G, et al. Integrating network toxicology and molecular docking to explore the toxicity of the environmental pollutant butyl hydroxyanisole: An example of induction of chronic urticaria. Heliyon. 2024;10(15):e35409. doi: 10.1016/j.heliyon.2024.e35409 39170477 PMC11336633

[16] Karaku F. Network toxicology for the cardiovascular toxicity analysis of tyrosine kinase inhibitors. Ankara Ecz Fak Derq. 2024;48(3):929. doi: 10.33483/jfpau.1478733

[17] Verleden SE, Hendriks JMH, Snoeckx A, Mai C, Mentens Y, Callebaut W, et al. Small Airway Disease in Pre-Chronic Obstructive Pulmonary Disease with Emphysema: A Cross-Sectional Study. Am J Respir Crit Care Med. 2024;209(6):683–92. doi: 10.1164/rccm.202301-0132OC 38055196

[18] Tang D, Chen M, Huang X, Zhang G, Zeng L, Zhang G, et al. SRplot: A free online platform for data visualization and graphing. PLoS One. 2023;18(11):e0294236. doi: 10.1371/journal.pone.0294236 37943830 PMC10635526

[19] Kanehisa M, Furumichi M, Sato Y, Matsuura Y, Ishiguro-Watanabe M. KEGG: biological systems database as a model of the real world. Nucleic Acids Res. 2025;53(D1):D672–7. doi: 10.1093/nar/gkae909 39417505 PMC11701520

[20] Kanehisa M, Furumichi M, Sato Y, Ishiguro-Watanabe M, Tanabe M. KEGG: integrating viruses and cellular organisms. Nucleic Acids Res. 2021;49(D1):D545–51. doi: 10.1093/nar/gkaa970 33125081 PMC7779016

[21] Kanehisa M, Goto S, Sato Y, Furumichi M, Tanabe M. KEGG for integration and interpretation of large-scale molecular data sets. Nucleic Acids Res. 2012;40(Database issue):D109–14. doi: 10.1093/nar/gkr988 22080510 PMC3245020

[22] Tian SJ, Liu ZY, Liu YS, Zeng JG. Research progress of pharmacokinetics in sanguinarine and chelerythrine. Heilongjiang Anim Sci Veterinary Med. 2017;(1):57–60. Chinese. doi: 10.13881/j.cnki.hljxmsy.20160615.001

[23] Lai GH, Cao JX, Zhou J, Wang F, Nie DR, Wen L, et al. Active ingredient and mechanism of Zhuanggu Zhentong capsule in the treatment of cancer-induced bone pain based on network pharmacology and experimental verification. Natural Prod Res Develop. 2024;36(12):2145–58. Chinese. doi: 10.16333/j.1001-6880.2024.12.015

[24] Deng YY, Dai YY, Wu YY, Li X, Zhu JH, Yu HH, et al. Investigating the mechanism of Rosa roxburghii Tratt. in atherosclerosis intervention through network pharmacology, UHPLC-Q Exactive, transcriptomics and molecular docking. Tradition Chinese Drug Res Clin Pharmacol. 2025;36(6):937–49. Chinese. doi: 10.19378/j.issn.1003-9783.2025.06.011

[25] Liu WN, Yu JX, Zhang HW, Jing JY, Tong JN, Zhang WS, et al. Exploring the mechanism of Gegen Qinlian Tang in ameliorating skeletal muscle insulin resistance based on transcriptomics. Chinese J Exp Tradition Med Formulae. 2025;36(6):1–18. Chinese. doi: 10.13422/j.cnki.syfjx.20250715

[26] Liang H, Li XY, Zhang XW, Bao YW, Ding ML, Yuan QH, et al. Mechanism of Ganke Granules intervening acute lung injury based on network pharmacology and experimental verification. China J Chinese Mater Med. 2024;49(8):2197–209. Chinese. doi: 10.19540/j.cnki.cjcmm.20240115.706 38812235

[27] Zhao SN, Li RH, Jia TZ. Study on content determination of 6 components in 21 batches of Chelidonium majus. Chinese Arch Tradition Chinese Med. 2020;38(11):207–9. Chinese. doi: 10.13193/j.issn.1673-7717.2020.11.050

[28] Zou X, Wang YM, Wang JQ, Long LL, Wang C, Liu Y, et al. Research progress on pharmacological activities of chelidonine. Drug Clinic. 2014;29(11):1326–30. Chinese.

[29] Zhou J, Qiu ZD, Wang YC, Xu JM, Huang XW. Research progress on chelerythrine. Ginseng Res. 2022;34(6):46–9. Chinese. doi: 10.19403/j.cnki.1671-1521.2022.06.013

[30] Zhao J, Zhang XL, He LM, Chen YL, Ming KH. Effects of Berberine on the Airway Inflammation of Rats with Chronic Obstructive Pulmonary Disease. China Medical Herald. 2015;12(31):40–3. Chinese.

[31] Yang F, Li X, Zhang TT, Li JR, Zhu YH, Hu YH, et al. Coptisine induces apoptosis in non-small cell lung cancer NCI-H1650 cells through ROS-dependent mitochondria pathway. J Pract Med. 2017;33(24):4033–7. Chinese.

[32] Zhang MY, Wang CL, Du XH, Huang S. The protective effect of sanguinarine on LPS-induced RAW264.7 cells through the STAT3 pathway. Tradition Chinese Drug Res Clin Pharmacol. 2017;28(6):714–8. Chinese. doi: 10.19378/j.issn.1003-9783.2017.06.003

[33] Liu TH, Yang ZS, Li N, Wu YY, Han SS, Han NP, et al. Study on chemical constituents of Feixi Tiaozhi formula based on UPLC-MS/MS. J Li-shizhen Tradition Chinese Med. 2020;31(1):36–8. Chinese.

[34] Hong B, Meng Q, Jiang JM, Zhu YJ, Xu YH, Zhao L. Research progress on alkaloids in Chelidonium majus L. Ginseng Res. 2022;34(2):58–62. Chinese. doi: 10.19403/j.cnki.1671-1521.2022.02.016

[35] Asadi M, Taghizadeh S, Kaviani E, Vakili O, Taheri-Anganeh M, Tahamtan M, et al. Caspase-3: Structure, function, and biotechnological aspects. Biotechnol Appl Biochem. 2022;69(4):1633–45. doi: 10.1002/bab.2233 34342377

[36] Solleti SK, Simon DM, Srisuma S, Arikan MC, Bhattacharya S, Rangasamy T, et al. Airway epithelial cell PPARγ modulates cigarette smoke-induced chemokine expression and emphysema susceptibility in mice. Am J Physiol Lung Cell Mol Physiol. 2015;309(3):L293-304. doi: 10.1152/ajplung.00287.2014 26024894 PMC4525123

[37] Mani S, Norel X, Varret M, Bchir S, Ben Anes A, Garrouch A, et al. Polymorphisms rs2745557 in PTGS2 and rs2075797 in PTGER2 are associated with the risk of chronic obstructive pulmonary disease development in a Tunisian cohort. Prostaglandins Leukot Essent Fatty Acids. 2021;166:102252. doi: 10.1016/j.plefa.2021.102252 33545665

[38] Naqvi KF, Mazzone SB, Shiloh MU. Infectious and Inflammatory Pathways to Cough. Annu Rev Physiol. 2023;85:71–91. doi: 10.1146/annurev-physiol-031422-092315 36170660 PMC9918720

[39] Liu YQ, Wang YF, Guo LJ. Changes of serum albumin concentration in type 2 diabetes mellitus and the influencing factors. J Chinese Pract Diag Therapy. 2019;33(11):1085–8. Chinese. doi: 10.13507/j.issn.1674-3474.2019.11.012

[40] Ezra A, Rabinovich-Nikitin I, Rabinovich-Toidman P, Solomon B. Multifunctional Effect of Human Serum Albumin Reduces Alzheimer’s Disease Related Pathologies in the 3xTg Mouse Model. J Alzheimers Dis. 2016;50(1):175–88. doi: 10.3233/JAD-150694 26682687

[41] Wu C-Y, Hu H-Y, Huang N, Chou Y-C, Li C-P, Chou Y-J. Albumin levels and cause-specific mortality in community-dwelling older adults. Prev Med. 2018;112:145–51. doi: 10.1016/j.ypmed.2018.04.015 29649489

[42] Wang L, Zhao H, Zhang L, Luo H, Chen Q, Zuo X. HSP90AA1, ADRB2, TBL1XR1 and HSPB1 are chronic obstructive pulmonary disease-related genes that facilitate squamous cell lung cancer progression. Oncol Lett. 2020;19(3):2115–22. doi: 10.3892/ol.2020.11318 32194709 PMC7039115

[43] Konings GFJ, Reynaert NL, Delvoux B, Verhamme FM, Bracke KR, Brusselle GG, et al. Increased levels of enzymes involved in local estradiol synthesis in chronic obstructive pulmonary disease. Mol Cell Endocrinol. 2017;443:23–31. doi: 10.1016/j.mce.2016.12.001 27940297

[44] Dinavahi SS, Nyayapathy S, Perumal Y, Dharmarajan S, Viswanadha S. Combined inhibition of PDE4 and PI3Kδ modulates the inflammatory component involved in the progression of chronic obstructive pulmonary disease. Drug Res (Stuttg). 2014;64(4):214–9. doi: 10.1055/s-0033-1355411 24105104

[45] Rajendrasozhan S, Yao H, Rahman I. Current perspectives on role of chromatin modifications and deacetylases in lung inflammation in COPD. COPD. 2009;6(4):291–7. doi: 10.1080/15412550903049132 19811389 PMC2760053

[46] Laddha AP, Kulkarni YA. VEGF and FGF-2: Promising targets for the treatment of respiratory disorders. Respir Med. 2019;156:33–46. doi: 10.1016/j.rmed.2019.08.003 31421589

[47] Kanazawa H, Asai K, Hirata K, Yoshikawa J. Possible effects of vascular endothelial growth factor in the pathogenesis of chronic obstructive pulmonary disease. Am J Med. 2003;114(5):354–8. doi: 10.1016/s0002-9343(02)01562-0 12714123

[48] Zhang LX, Tian YG, Dong HR, Chen K, Shao D, Zhao GY, et al. Mechanism study on effective-component compatibility of Bufei Yishen formula inhibiting pulmonary vascular remodeling with chronic obstructive pulmonary disease via VEGF/PI3K/Akt pathway. Chinese J Integrated Tradition West Med. 2022;42(11):1356–62. Chinese. doi: 10.19656/j.cnki.1002-2406.210314

[49] Wu Y, Shi SF. Potential molecular biological mechanism of TCM in preventing and treating airway remodeling of COPD. Inf Tradition Chinese Med. 2021;38(3):69–72. Chinese. doi: 10.19656/j.cnki.1002-2406.210314

[50] Xie WY, Bao YS, Wang JY, Shang LZ, Wang XY, Li L, et al. Signal pathways relating to prevention and treatment of airway remodeling in chronic obstructive pulmonary disease with traditional Chinese medicine. Chinese J Exp Tradition Med Formulae. 2019;25(23):1–8. Chinese. doi: 10.13422/j.cnki.syfjx.20191307

[51] Rainer PP, Kass DA. Old dog, new tricks: novel cardiac targets and stress regulation by protein kinase G. Cardiovasc Res. 2016;111(2):154–62. doi: 10.1093/cvr/cvw107 27297890 PMC4937204

[52] Liu WH, Xia XL, Xia HJ. Based on the PI3K/AKT/mTOR signaling pathway, the research progress of traditional Chinese medicine in the prevention and treatment of COPD was discussed. J Pract Tradition Chinese Intern Med. 2025;39(6):87–90. Chinese. doi: 10.13729/j.issn.1671-7813.Z20240918006

[53] Liang KK, Wang ZW, Huang KT, Zhao Y, Li JY, Du Y, et al. Traditional Chinese medicine regulates airway remodeling of chronic obstructive pulmonary disease via PI3K-AKT signaling pathway: a review. Pharmacol Clin Chinese Mater Med. 2024;40(5):103–8. Chinese. doi: 10.13412/j.cnki.zyyl.20230725.004

[54] Bao BB, Zhang P, Xu BC, Zhang YY, Li SY, Xie Y. Analysis of animal model of chronic obstructive pulmonary disease based on clinical characteristics of traditional Chinese and Western medicine. Chinese J Exp Tradition Med Formulae. 2025;36(6):1–23. Chinese. doi: 10.13422/j.cnki.syfjx.20250543

[55] Hou HP, Zhang GP, Li H, Chen TF, Gao YH, Song L, et al. Establishment of chronic obstructive pulmonary disease cell model in vitro by stimulating A549 and RAW264.7 cells with CSE and LPS. J Guangxi Med Univ. 2020;37(10):1772–7. Chinese. doi: 10.16190/j.cnki.45-1211/r.2020.10.003

[56] Zhang HX, Wang X, Liu YP, Di X. Metabolic kinetic and subtypes of CYP450 of chelidonine in rat liver microsomal enzyme. J Shenyang Pharmaceutical Univ. 2015;32(4):276–80. Chinese. doi: 10.14066/j.cnki.cn21-1349/r.2015.04.006

[57] Wu C, Wang X, Xu M, Liu Y, Di X. Intracellular Accumulation as an Indicator of Cytotoxicity to Screen Hepatotoxic Components of Chelidonium majus L. by LC-MS/MS. Molecules. 2019;24(13):2410. doi: 10.3390/molecules24132410 31261913 PMC6651743

[58] Kurokawa T, Shimomura Y, Bajotto G, Kotake K, Arikawa T, Ito N, et al. Peroxisome proliferator-activated receptor α (PPARα) mRNA expression in human hepatocellular carcinoma tissue and non-cancerous liver tissue. World J Surg Oncol. 2011;9:167. doi: 10.1186/1477-7819-9-167 22168458 PMC3260121

[59] McGreal SR, Rumi K, Soares MJ, Woolbright BL, Jaeschke H, Apte U. Disruption of Estrogen Receptor Alpha in Rats Results in Faster Initiation of Compensatory Regeneration Despite Higher Liver Injury After Carbon Tetrachloride Treatment. Int J Toxicol. 2017;36(3):199–206. doi: 10.1177/1091581817706067 28481132 PMC5535772

[60] Habib SA, Abdelrahman RS, Abdel Rahim M, Suddek GM. Anti-apoptotic effect of vinpocetine on cisplatin-induced hepatotoxicity in mice: The role of Annexin-V, Caspase-3, and Bax. J Biochem Mol Toxicol. 2020;34(10):e22555. doi: 10.1002/jbt.22555 32578916

[61] Hu Y, Yang XF, Wu SD, Xiao JH. COX-2 in liver fibrosis. Clin Chim Acta. 2020;506:196–203. doi: 10.1016/j.cca.2020.03.024 32184095

[62] Gao LY, Liang BY, Jin C. Role of angiogenesis in the development and progression of liver diseases. Progr Physiol Sci. 2020;51(3):193–7. Chinese.

[63] Lu T, Yang X, Shi Y, Zhao M, Bi G, Liang J, et al. Single-cell transcriptome atlas of lung adenocarcinoma featured with ground glass nodules. Cell Discov. 2020;6:69. doi: 10.1038/s41421-020-00200-x 33083004 PMC7536439

[64] Coothankandaswamy V, Liu Y, Mao S-C, Morgan JB, Mahdi F, Jekabsons MB, et al. The alternative medicine pawpaw and its acetogenin constituents suppress tumor angiogenesis via the HIF-1/VEGF pathway. J Nat Prod. 2010;73(5):956–61. doi: 10.1021/np100228d 20423107 PMC2890309

[65] Lakshmikanthan S, Sobczak M, Chun C, Henschel A, Dargatz J, Ramchandran R, et al. Rap1 promotes VEGFR2 activation and angiogenesis by a mechanism involving integrin αvβ₃. Blood. 2011;118(7):2015–26. doi: 10.1182/blood-2011-04-349282 21636859 PMC3158727

[66] Zhang JJ, Pu Y, Li Y, Shen CY, Zhang XM. Influence of PI3K and MAPKs signaling pathways on expression of VEGF in human pancreatic cancer PANC-1 cells. J North Sichuan Med Coll. 2014;29(1):44–8. Chinese.

[67] Wu CH, Shen M, Li L. Association between PI3K/Akt/mTOR/p70^S6K^ signaling pathway and hepatic fibrosis. J Clin Hepat. 2015;31(11):1928–32. Chinese.

[68] El-Boshy M, Refaat B, Almaimani RA, Abdelghany AH, Ahmad J, Idris S, et al. Vitamin D3 and calcium cosupplementation alleviates cadmium hepatotoxicity in the rat: Enhanced antioxidative and anti-inflammatory actions by remodeling cellular calcium pathways. J Biochem Mol Toxicol. 2020;34(3):e22440. doi: 10.1002/jbt.22440 31926057

[69] Cheng R, Du J, Wu W, Jiang ZZ, Zhang LY, Huang X. Effects of seven traditional Chinese medicine monomers on Oct1 and Oatp1b2 transporters in rat primary hepatocytes. Herald Med. 2020;39(4):562–7. Chinese.

[70] Li LM, Zang QC, Zhang RP, Abliz Z. Mass spectrometry-based metabolomics in the study of in vitro drug hepatotoxicity evaluation. J Chinese Mass Spectr Soc. 2021;42(5):772–86. Chinese.

[71] Zhao SM, Wang Y, Liu C, Cui SF. Preparation of animal models of drug-induced liver injury. Acta Laborator Anim Sci Sinica. 2023;31(7):935–45. Chinese.

[72] Hu YY, Sun ZW. Synergistic and integrated mechanism of traditional Chinese medicine compound prescriptions in respiratory diseases. J Tradition Chinese Med Manag. 2025;33(3):91–3. Chinese. doi: 10.16690/j.cnki.1007-9203.2025.03.008

[73] Zhao MM, Miao MS. Toxicity Reduction Measures of Herbal Medicines Causing Liver Injury and Role of Herbal Medicines in Preventing and Treating Liver Injury. Chinese J Exp Tradition Med Formulae. 2023;29(15):273–82. Chinese. doi: 10.13422/j.cnki.syfjx.20230711

[74] Lou X, Tian S, Bai M, Miao YY, Miao MS. A new toxicity grading method of toxic traditional Chinese medicine——Ⅳ grade toxicity classification. Acta Chinese Med. 2020;35(2):370–3. Chinese. doi: 10.16368/j.issn.1674-8999.2020.02.084

[75] Du HT, Wang L, Ding J, Du YX, Wang P. Application status and challenges of molecular docking in development of traditional Chinese medicine. China J Chinese Mater Med. 2024;49(3):671–80. Chinese. doi: 10.19540/j.cnki.cjcmm.20231013.703 38621871

[76] Mao LS, Zhu XH. Application progress of network pharmacology in traditional Chinese medicine. J Tradition Chinese Med Manag. 2021;29(13):98–102. Chinese. doi: 10.16690/j.cnki.1007-9203.2021.13.040

[77] Zhang Q, Chang J, Ji WW, Zheng H, Xiang YY, Liu LH, et al. Research progress of network pharmacology in the TCM field. Chinese J Inf Tradition Chinese Med. 2024;31(11):186–90. Chinese. doi: 10.19879/j.cnki.1005-5304.202207685

