# Supplementary figures and images for: Dual efficacy-toxicity of Chelidonii Herba in chronic obstructive pulmonary disease: Integrated network pharmacology, immune profiling and molecular docking
Source: PLoS One. 2025 Sep 23;20(9):e0332750. doi: 10.1371/journal.pone.0332750 (PMC12456800; doi:10.1371/journal.pone.0332750)

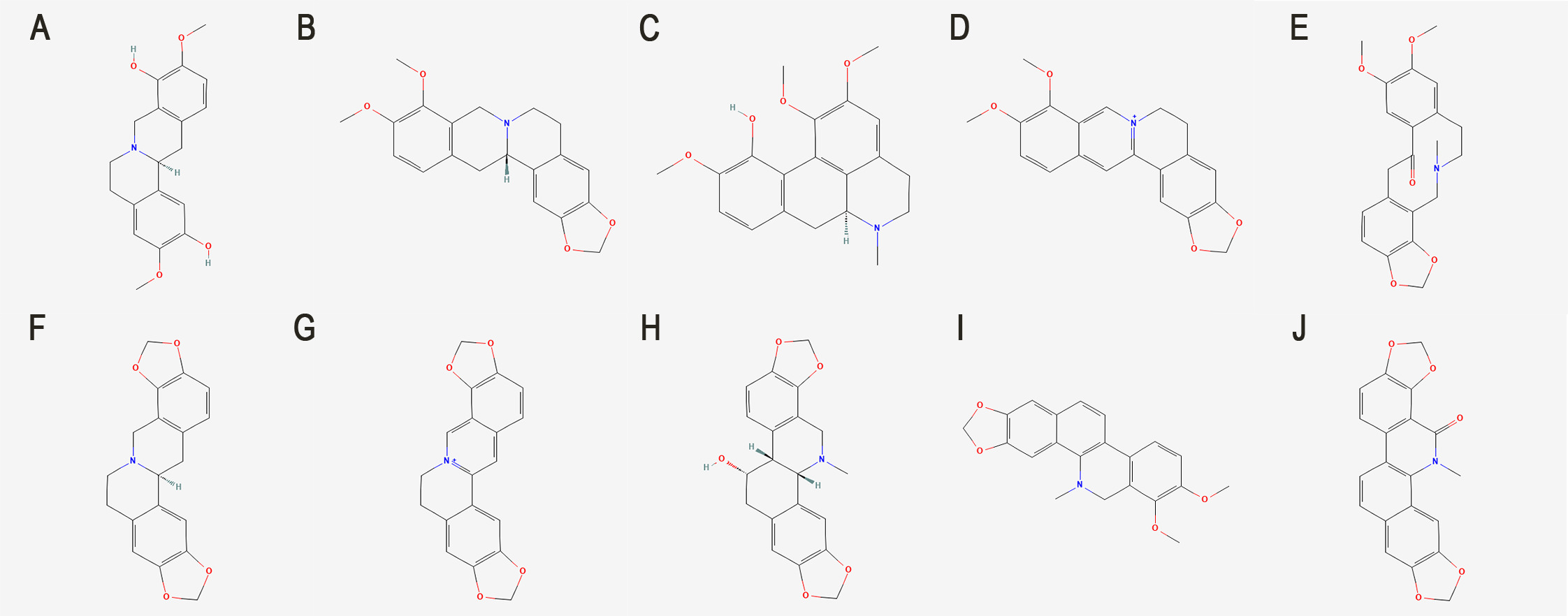

Supplement: S1 Fig — Chemical structures were sourced from the PubChem database (https://pubchem.ncbi.nlm.nih.gov/). Panel designations correspond to Table 2 entries: (A) (S)-Scoulerine; (B) (S)-Canadine; (C) Luteanin; (D) berberine; (E) Cryptopin; (F) (S)-Stylopine; (G) coptisine; (H) chelidonine; (I) Dihydrochelerythrine; (J) Oxysanguinarine. (TIF) [file pone.0332750.s001.tif]

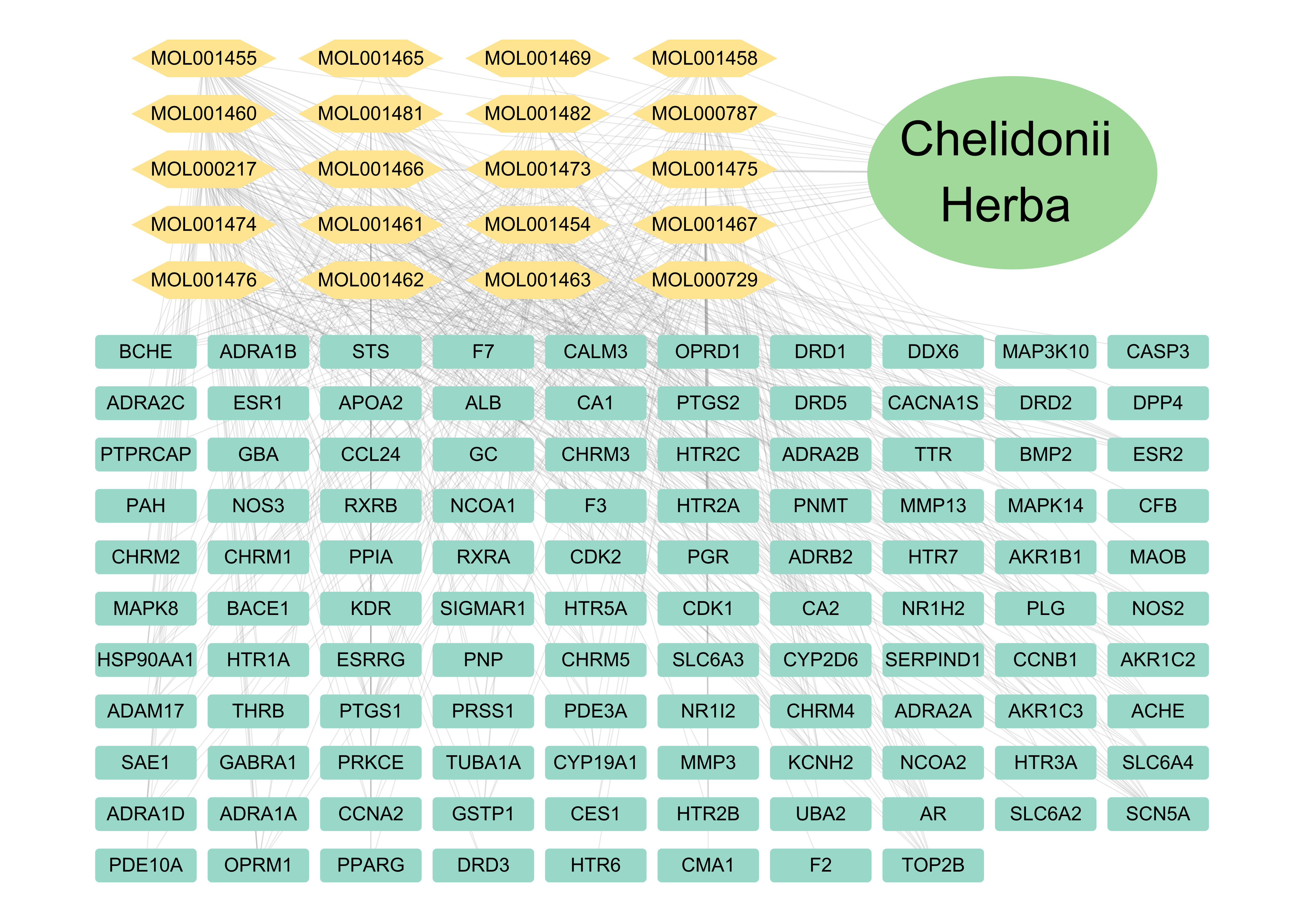

Supplement: S2 Data — (ZIP) [file pone.0332750.s003.zip › 2.Drug_Component_Target_Network/network.txt.png]

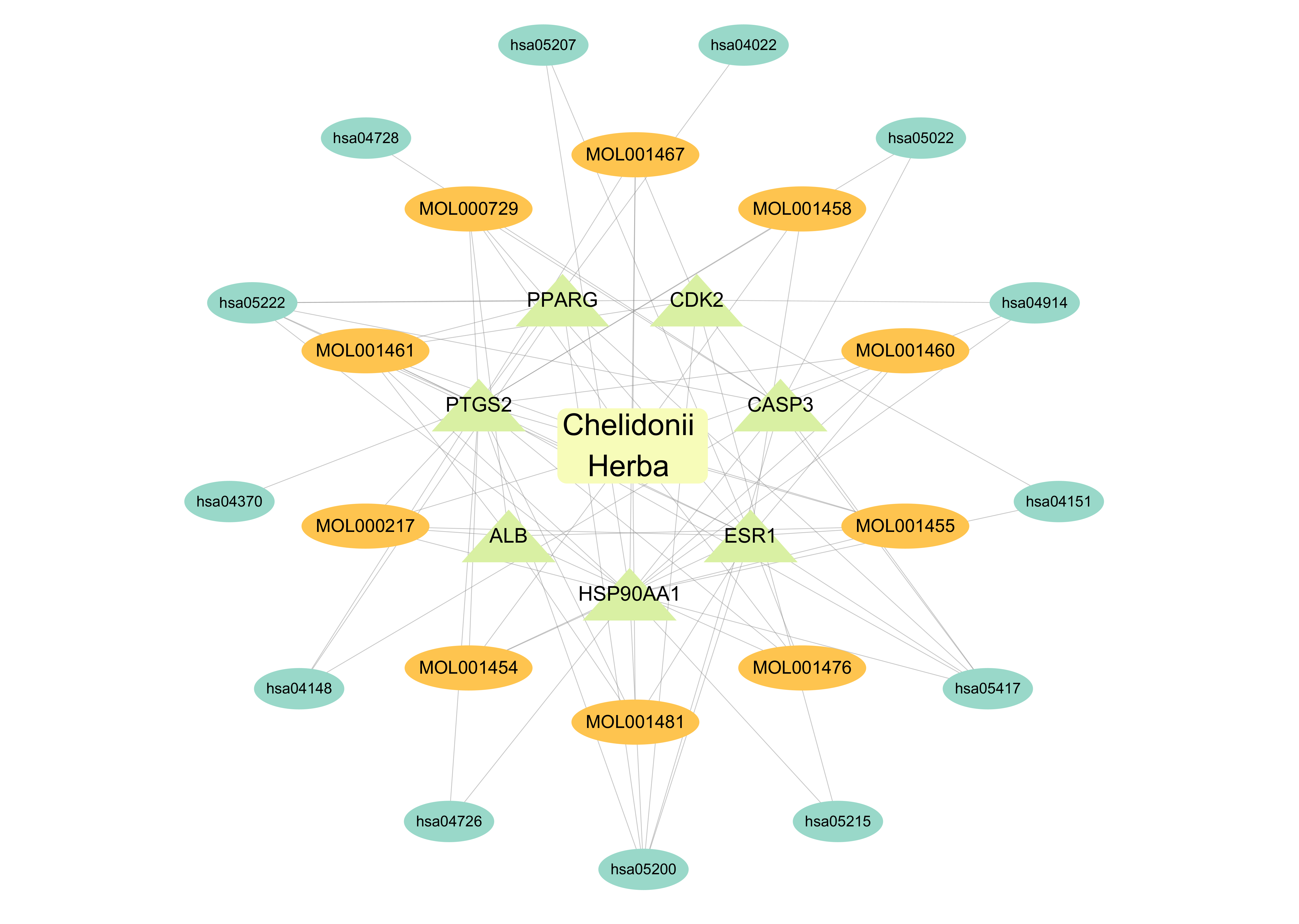

Supplement: S7 Data — (ZIP) [file pone.0332750.s008.zip › 7.Drug_Component_CoreTarget_Pathway/白屈菜-COPD/network.txt.png]

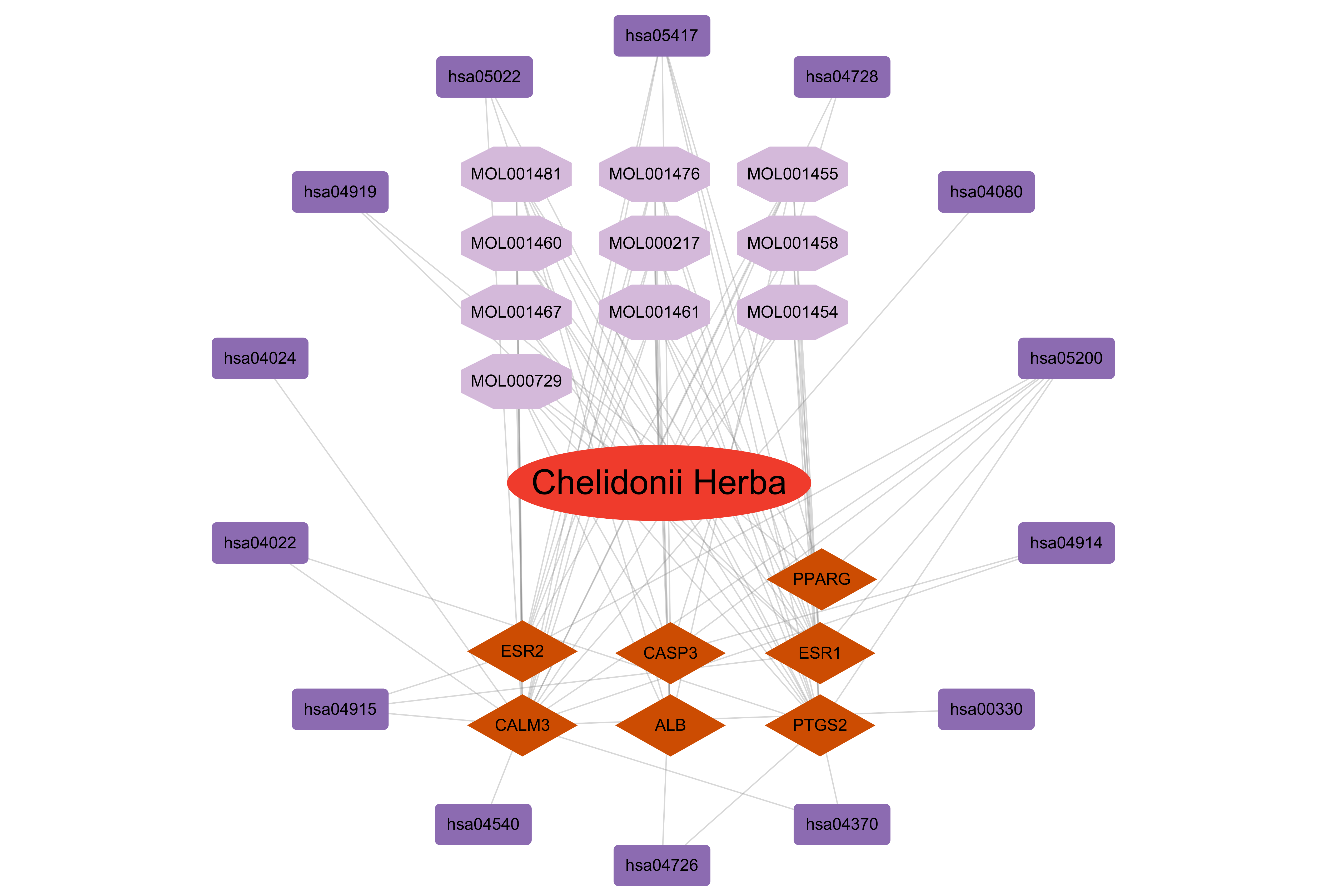

Supplement: S7 Data — (ZIP) [file pone.0332750.s008.zip › 7.Drug_Component_CoreTarget_Pathway/白屈菜-Drug Induce Liver Injury/network.txt.png]

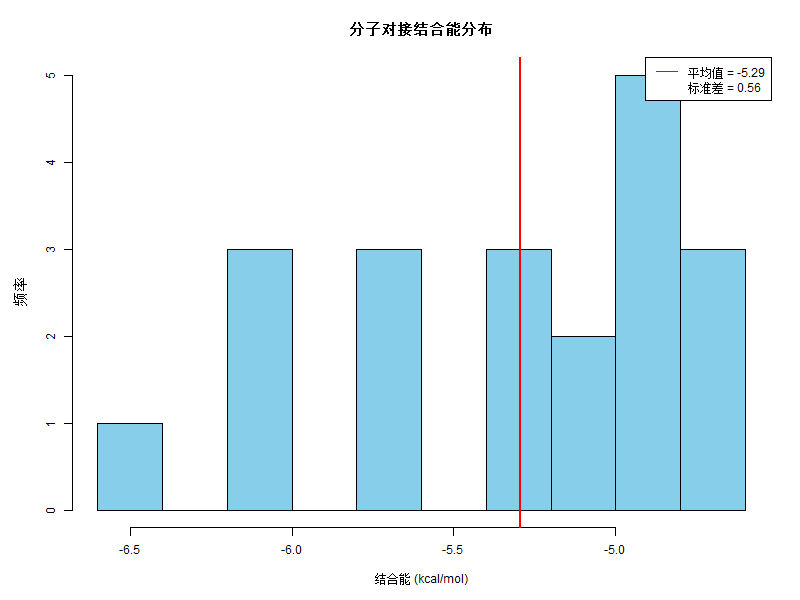

Supplement: S8 Data — (ZIP) [file pone.0332750.s009.zip › 8.Molecular_Docking/对接结果分析/(S)-Canadine-CALM3/docking_energy_distribution.png]

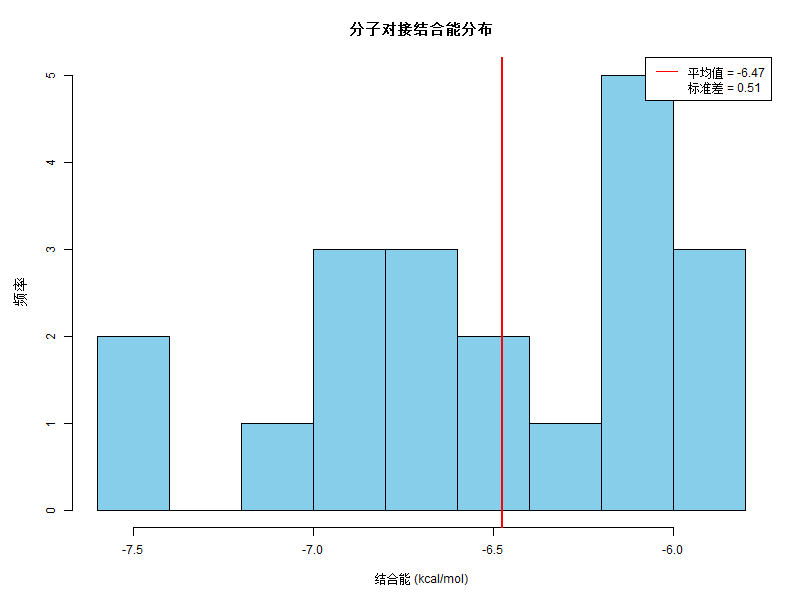

Supplement: S8 Data — (ZIP) [file pone.0332750.s009.zip › 8.Molecular_Docking/对接结果分析/(S)-Canadine-CASP3/docking_energy_distribution.png]

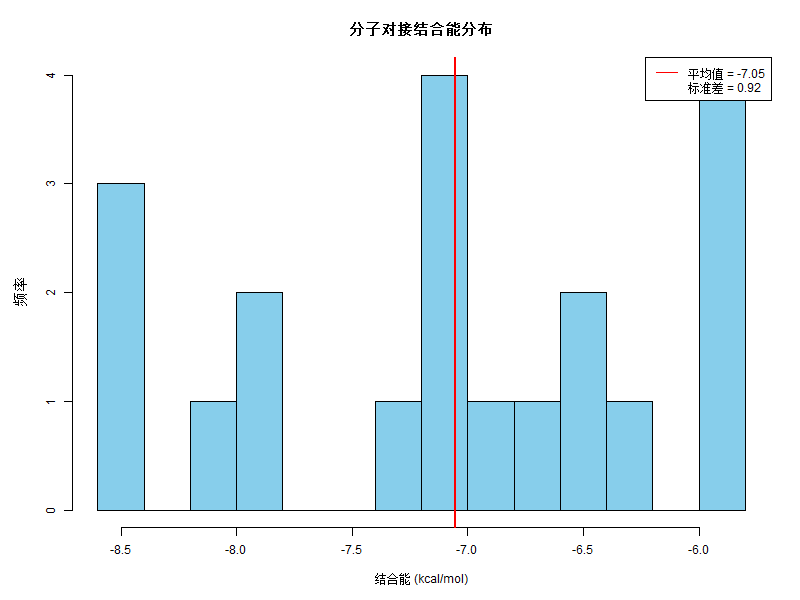

Supplement: S8 Data — (ZIP) [file pone.0332750.s009.zip › 8.Molecular_Docking/对接结果分析/(S)-Canadine-CDK2/docking_energy_distribution.png]

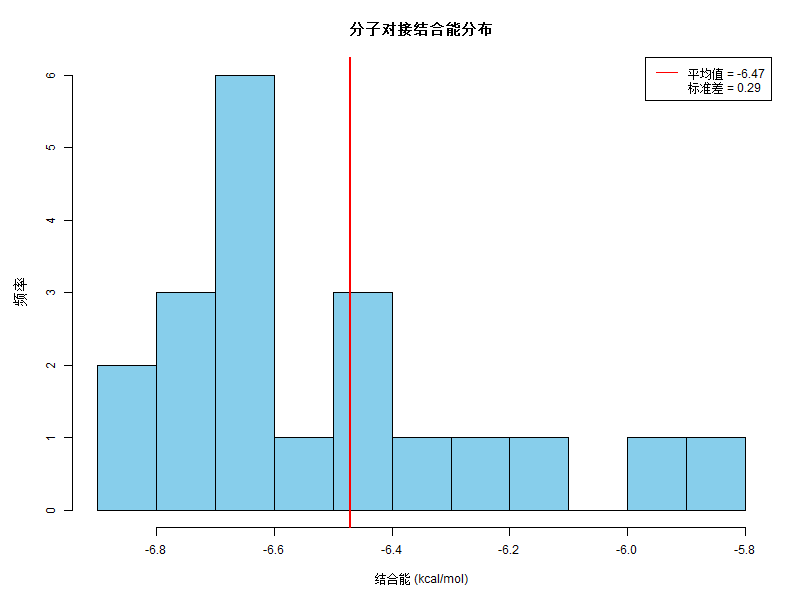

Supplement: S8 Data — (ZIP) [file pone.0332750.s009.zip › 8.Molecular_Docking/对接结果分析/(S)-Canadine-ESR1/docking_energy_distribution.png]

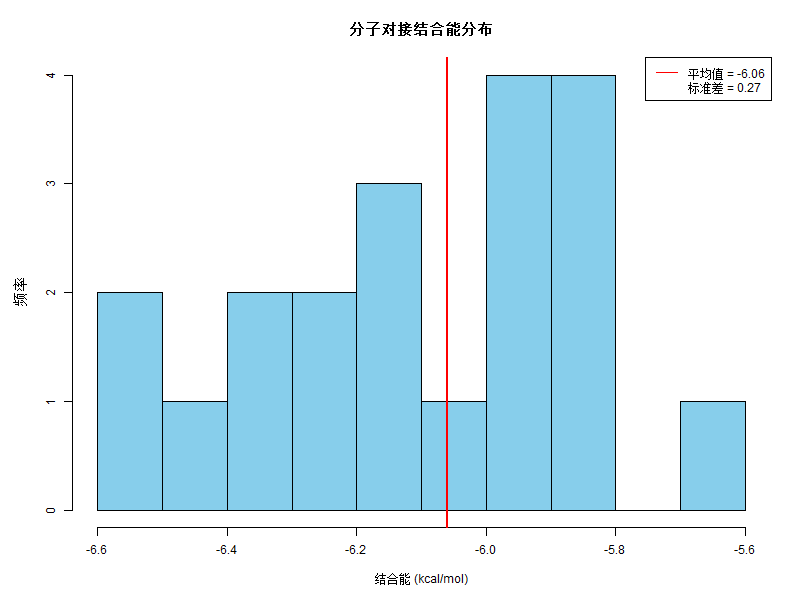

Supplement: S8 Data — (ZIP) [file pone.0332750.s009.zip › 8.Molecular_Docking/对接结果分析/(S)-Canadine-ESR2/docking_energy_distribution.png]

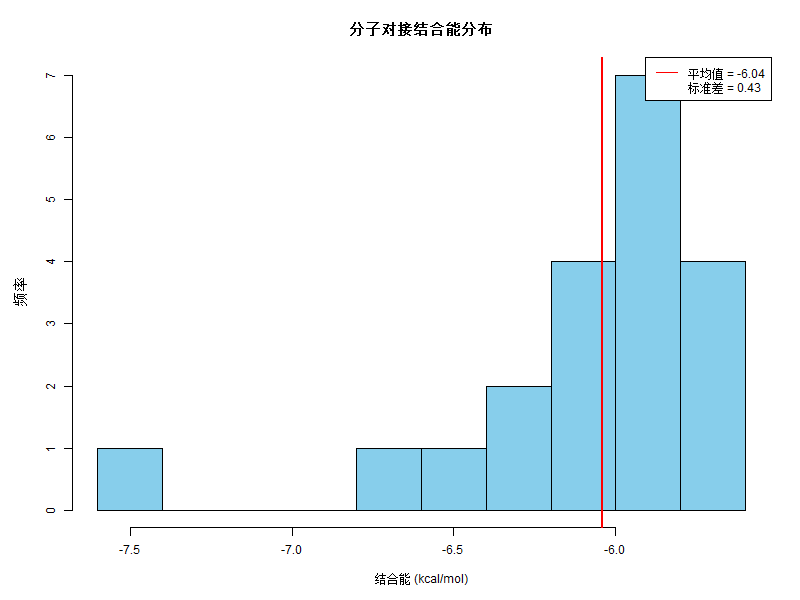

Supplement: S8 Data — (ZIP) [file pone.0332750.s009.zip › 8.Molecular_Docking/对接结果分析/(S)-Canadine-HSP90AA1/docking_energy_distribution.png]

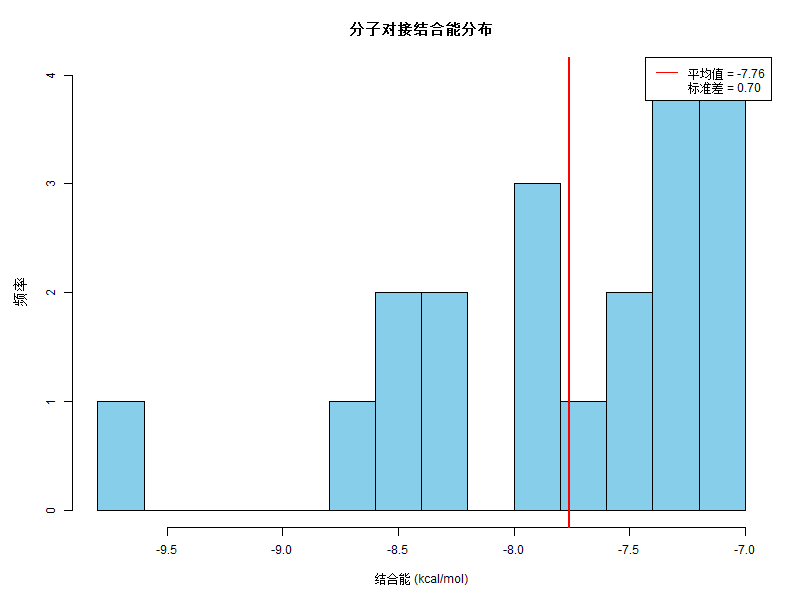

Supplement: S8 Data — (ZIP) [file pone.0332750.s009.zip › 8.Molecular_Docking/对接结果分析/(S)-Canadine-PTGS2/docking_energy_distribution.png]

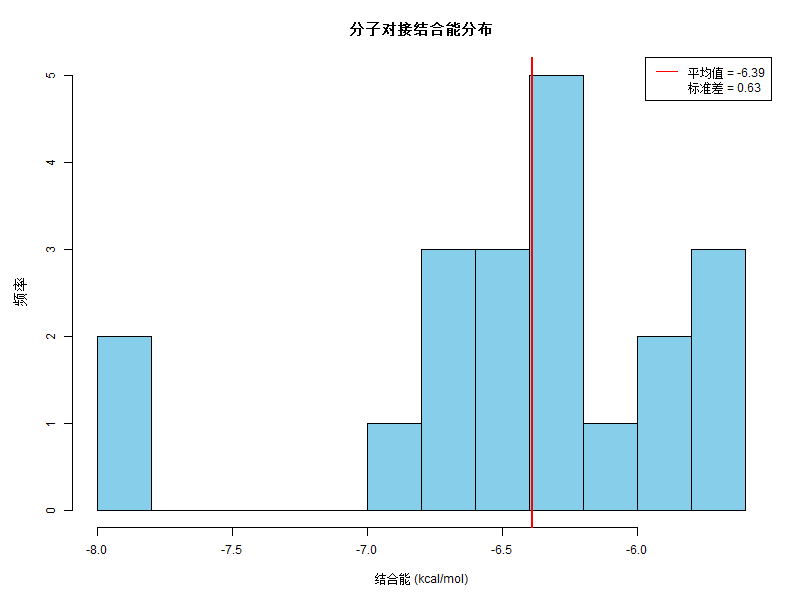

Supplement: S8 Data — (ZIP) [file pone.0332750.s009.zip › 8.Molecular_Docking/对接结果分析/(S)-Scoulerine-CASP3/docking_energy_distribution.png]

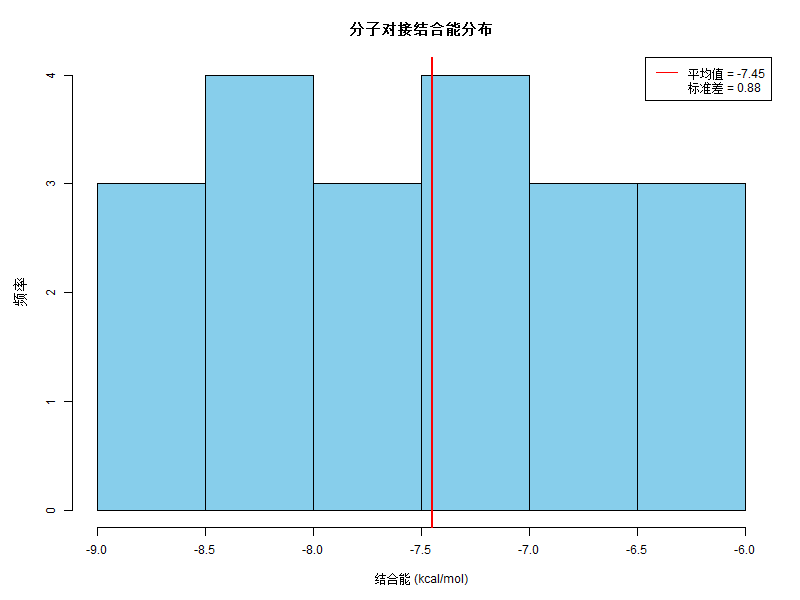

Supplement: S8 Data — (ZIP) [file pone.0332750.s009.zip › 8.Molecular_Docking/对接结果分析/(S)-Scoulerine-CDK2/docking_energy_distribution.png]

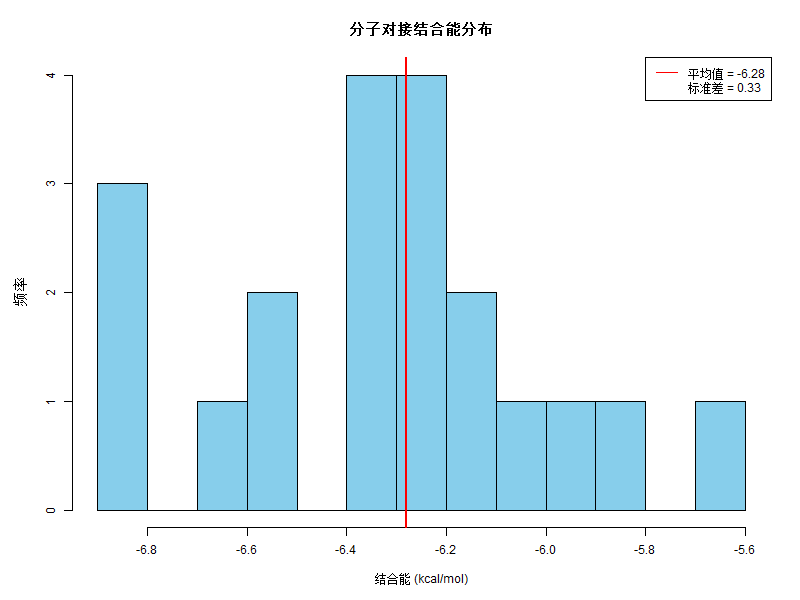

Supplement: S8 Data — (ZIP) [file pone.0332750.s009.zip › 8.Molecular_Docking/对接结果分析/(S)-Scoulerine-ESR1/docking_energy_distribution.png]

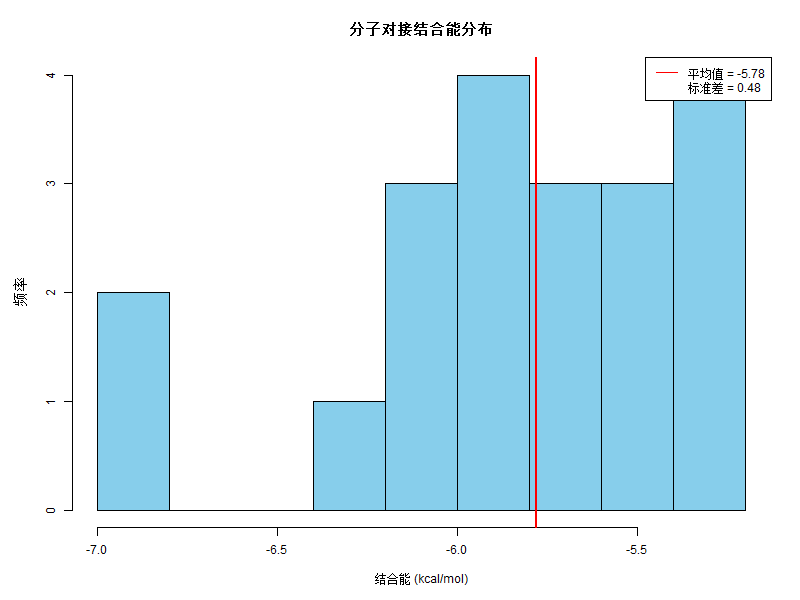

Supplement: S8 Data — (ZIP) [file pone.0332750.s009.zip › 8.Molecular_Docking/对接结果分析/(S)-Scoulerine-HSP90AA1/docking_energy_distribution.png]

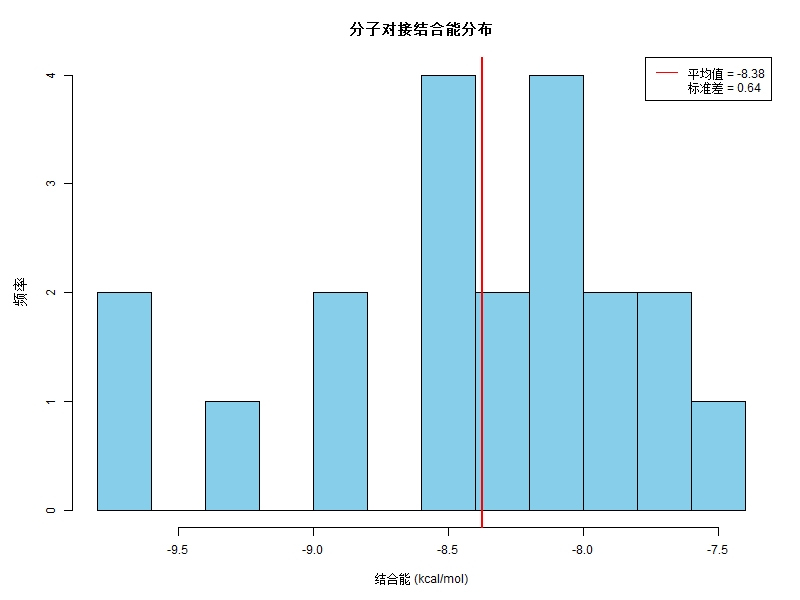

Supplement: S8 Data — (ZIP) [file pone.0332750.s009.zip › 8.Molecular_Docking/对接结果分析/(S)-Scoulerine-PTGS2/docking_energy_distribution.png]

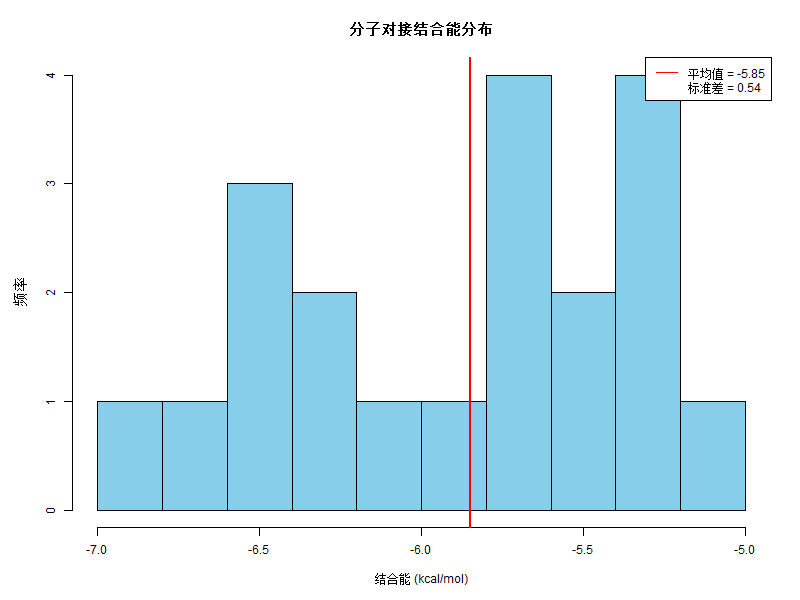

Supplement: S8 Data — (ZIP) [file pone.0332750.s009.zip › 8.Molecular_Docking/对接结果分析/(S)-Stylopine-CALM3/docking_energy_distribution.png]

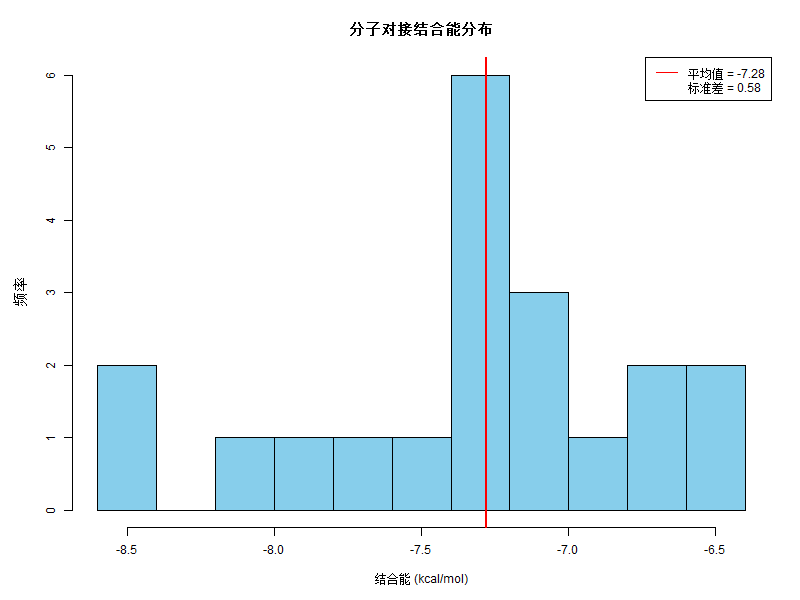

Supplement: S8 Data — (ZIP) [file pone.0332750.s009.zip › 8.Molecular_Docking/对接结果分析/(S)-Stylopine-CASP3/docking_energy_distribution.png]

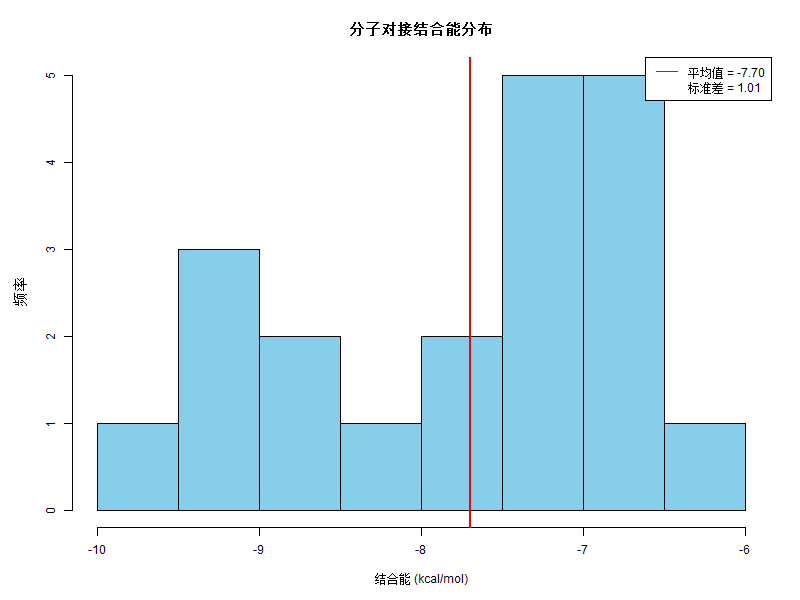

Supplement: S8 Data — (ZIP) [file pone.0332750.s009.zip › 8.Molecular_Docking/对接结果分析/(S)-Stylopine-CDK2/docking_energy_distribution.png]

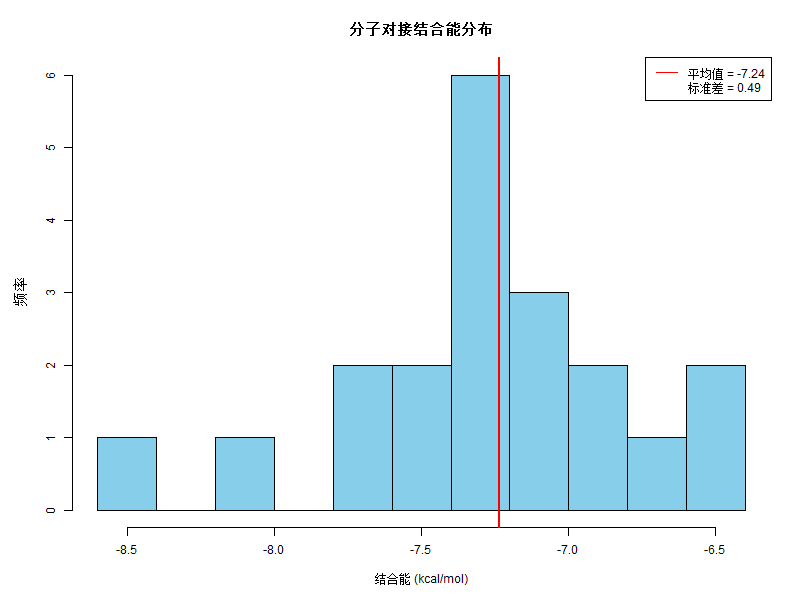

Supplement: S8 Data — (ZIP) [file pone.0332750.s009.zip › 8.Molecular_Docking/对接结果分析/(S)-Stylopine-ESR1/docking_energy_distribution.png]

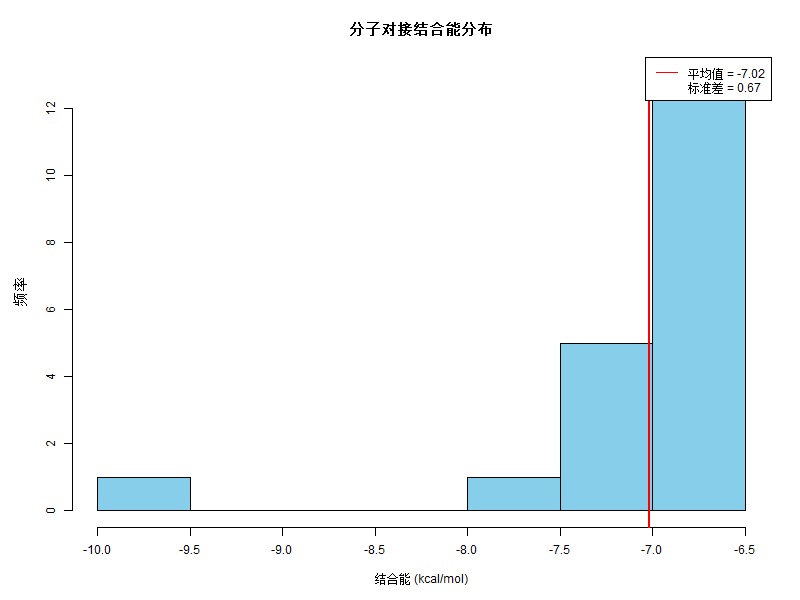

Supplement: S8 Data — (ZIP) [file pone.0332750.s009.zip › 8.Molecular_Docking/对接结果分析/(S)-Stylopine-ESR2/docking_energy_distribution.png]

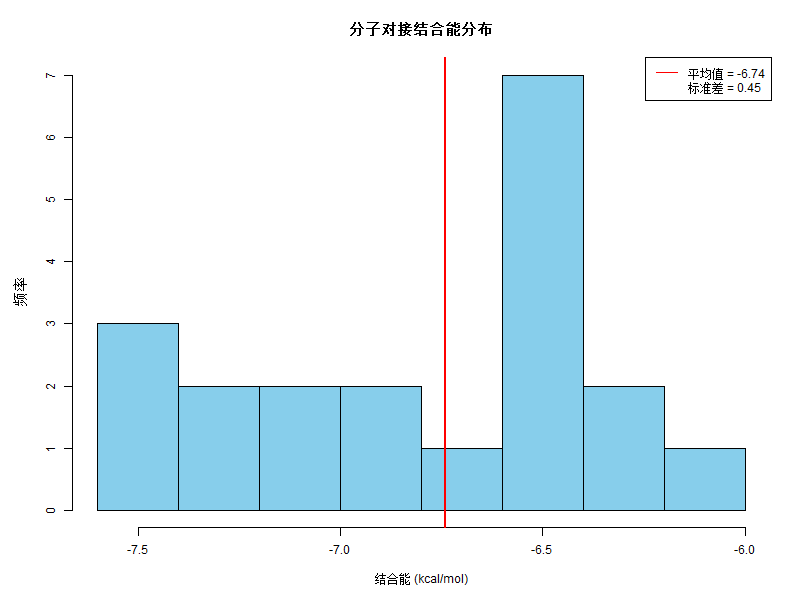

Supplement: S8 Data — (ZIP) [file pone.0332750.s009.zip › 8.Molecular_Docking/对接结果分析/(S)-Stylopine-HSP90AA1/docking_energy_distribution.png]

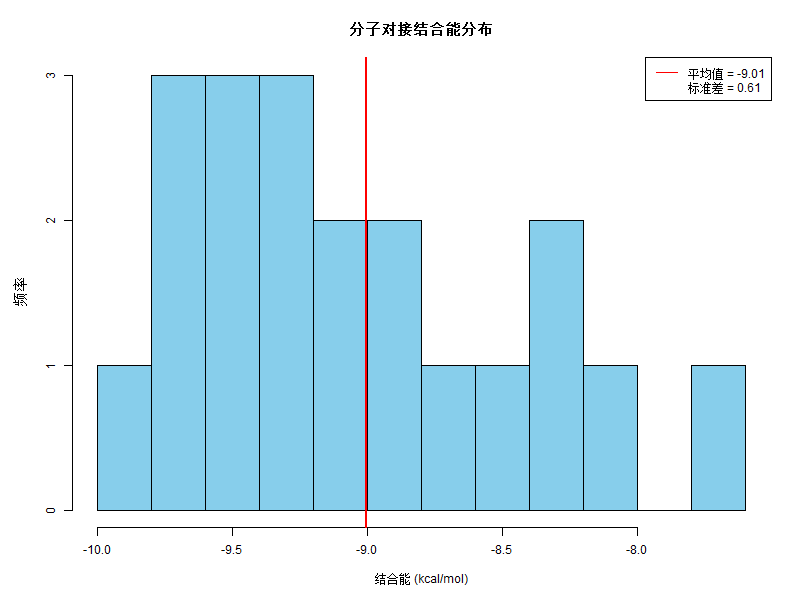

Supplement: S8 Data — (ZIP) [file pone.0332750.s009.zip › 8.Molecular_Docking/对接结果分析/(S)-Stylopine-PTGS2/docking_energy_distribution.png]

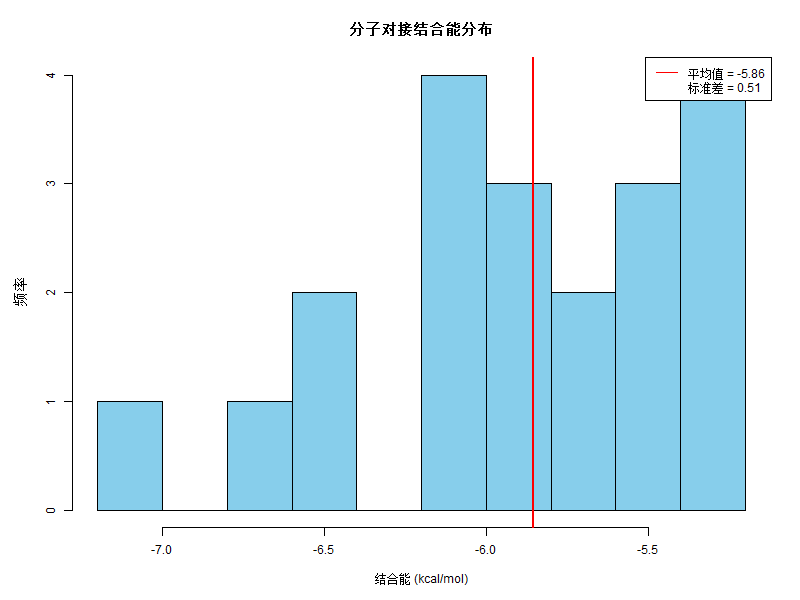

Supplement: S8 Data — (ZIP) [file pone.0332750.s009.zip › 8.Molecular_Docking/对接结果分析/Chelidonine-CALM3/docking_energy_distribution.png]

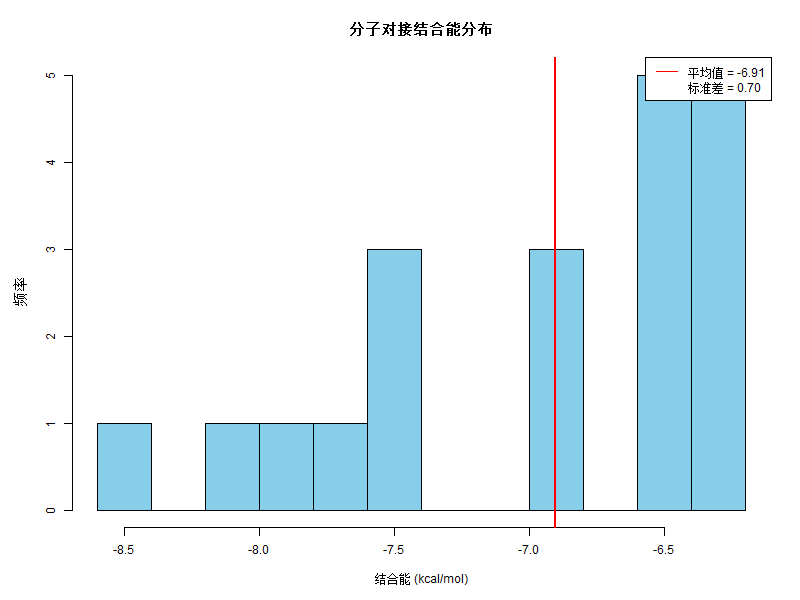

Supplement: S8 Data — (ZIP) [file pone.0332750.s009.zip › 8.Molecular_Docking/对接结果分析/Chelidonine-CASP3/docking_energy_distribution.png]

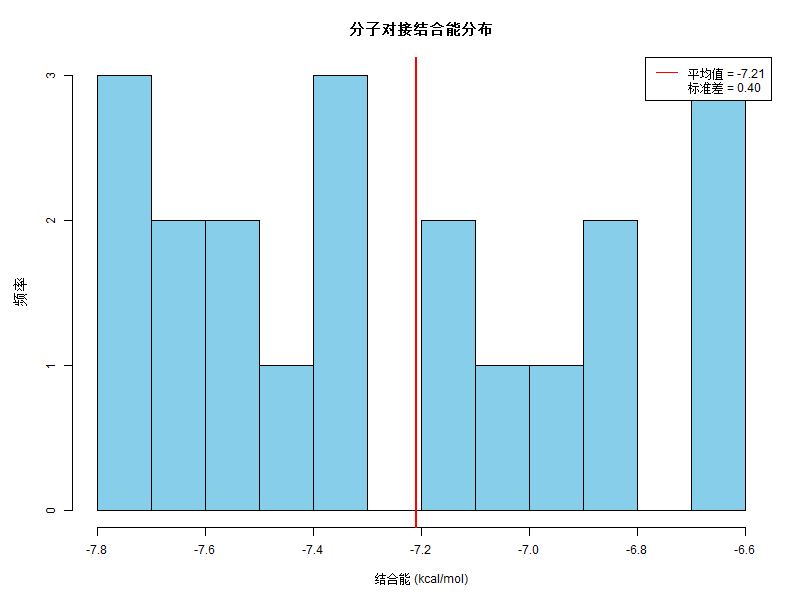

Supplement: S8 Data — (ZIP) [file pone.0332750.s009.zip › 8.Molecular_Docking/对接结果分析/Chelidonine-ESR1/docking_energy_distribution.png]

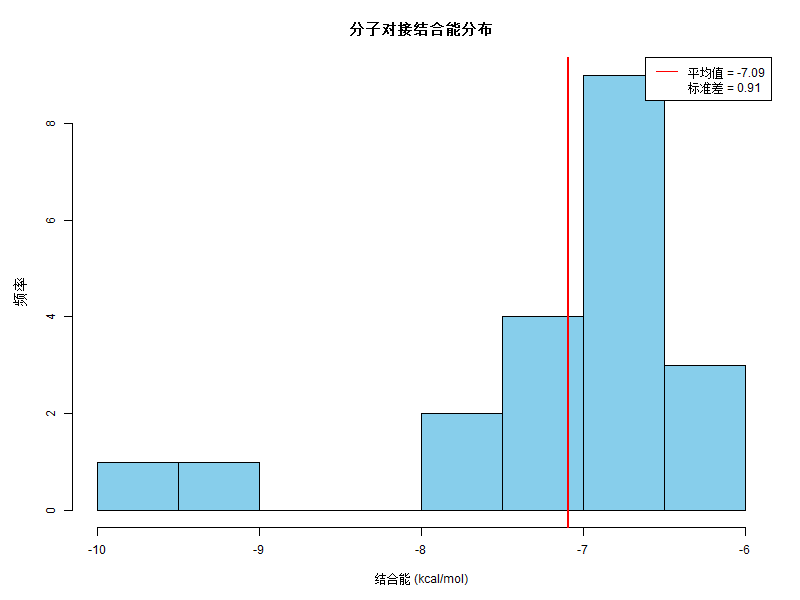

Supplement: S8 Data — (ZIP) [file pone.0332750.s009.zip › 8.Molecular_Docking/对接结果分析/Chelidonine-ESR2/docking_energy_distribution.png]

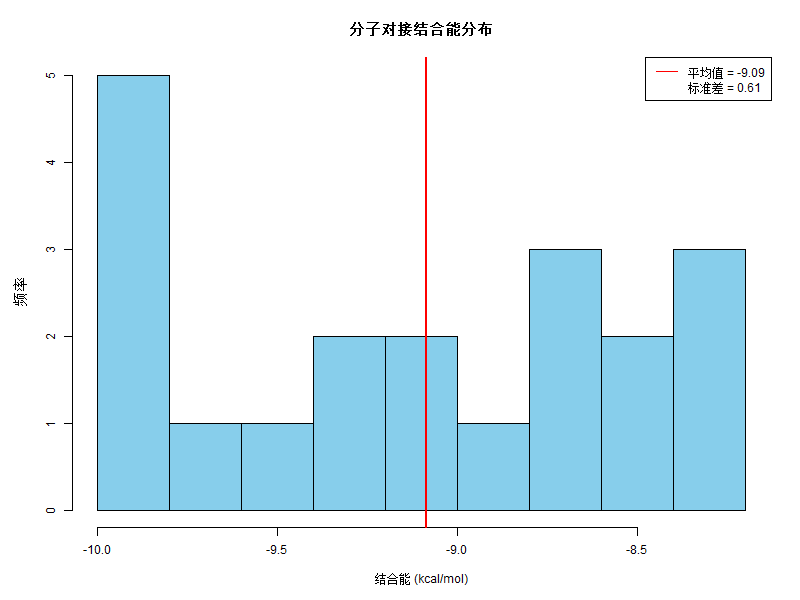

Supplement: S8 Data — (ZIP) [file pone.0332750.s009.zip › 8.Molecular_Docking/对接结果分析/Chelidonine-PTGS2/docking_energy_distribution.png]

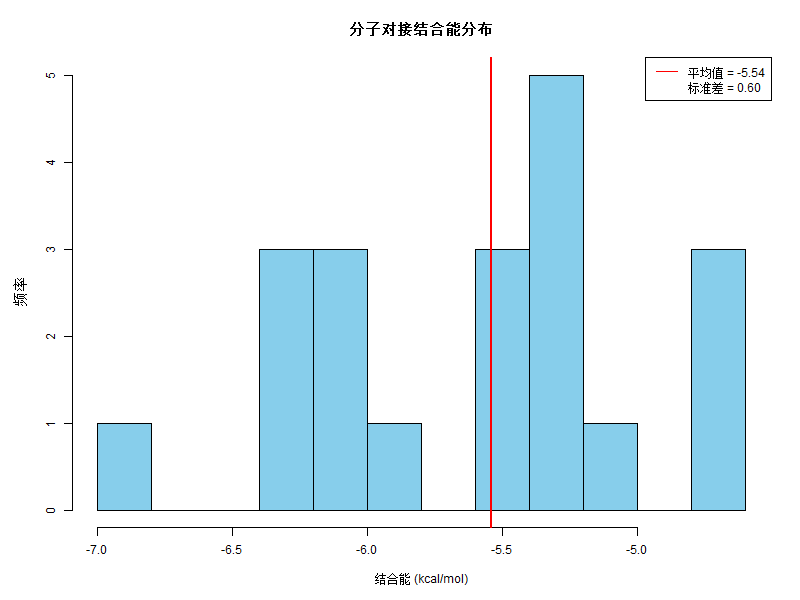

Supplement: S8 Data — (ZIP) [file pone.0332750.s009.zip › 8.Molecular_Docking/对接结果分析/Dihydrochelerythrine-CALM3/docking_energy_distribution.png]

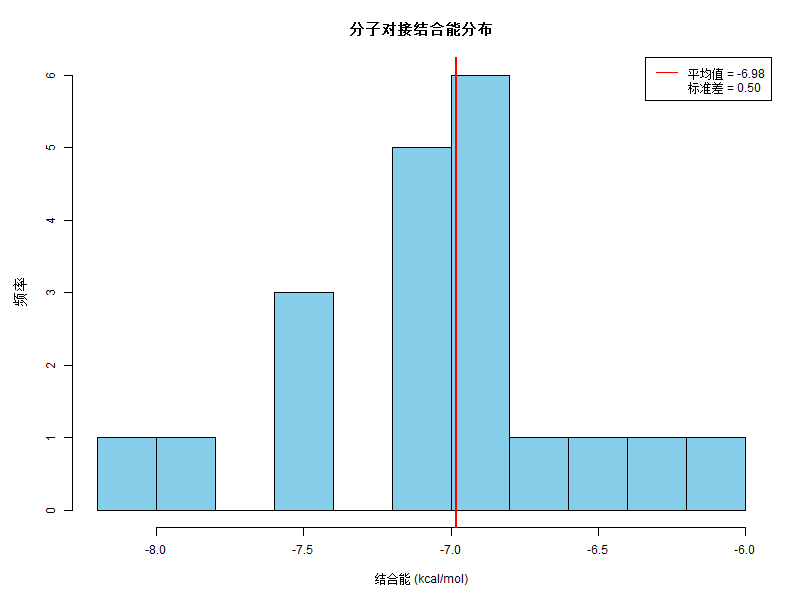

Supplement: S8 Data — (ZIP) [file pone.0332750.s009.zip › 8.Molecular_Docking/对接结果分析/Dihydrochelerythrine-CASP3/docking_energy_distribution.png]

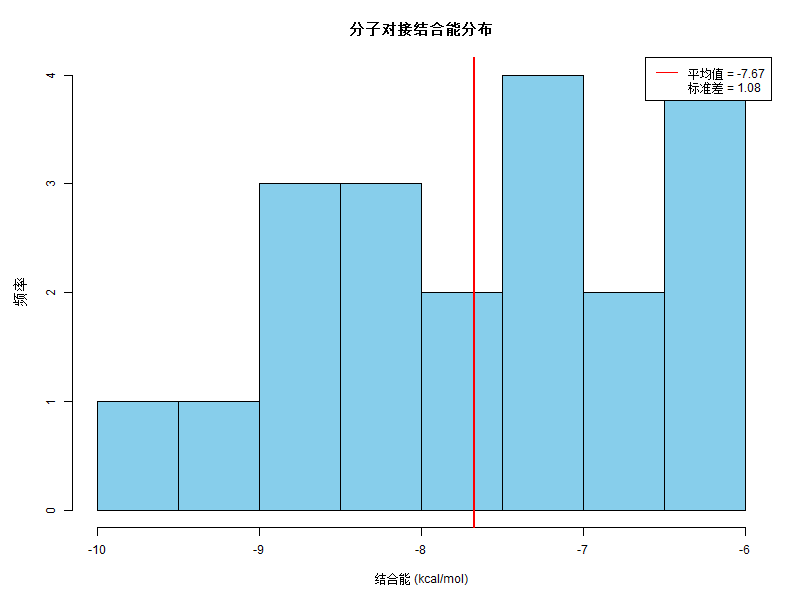

Supplement: S8 Data — (ZIP) [file pone.0332750.s009.zip › 8.Molecular_Docking/对接结果分析/Dihydrochelerythrine-CDK2/docking_energy_distribution.png]

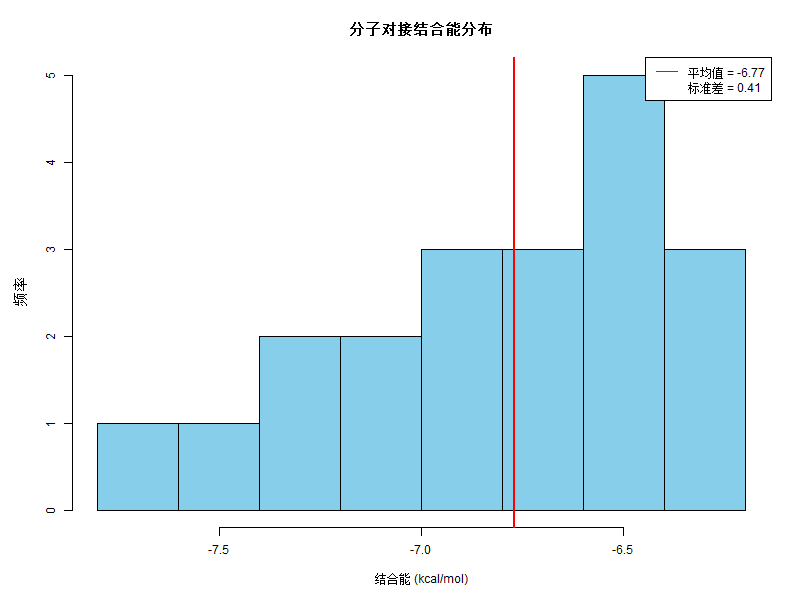

Supplement: S8 Data — (ZIP) [file pone.0332750.s009.zip › 8.Molecular_Docking/对接结果分析/Dihydrochelerythrine-ESR1/docking_energy_distribution.png]

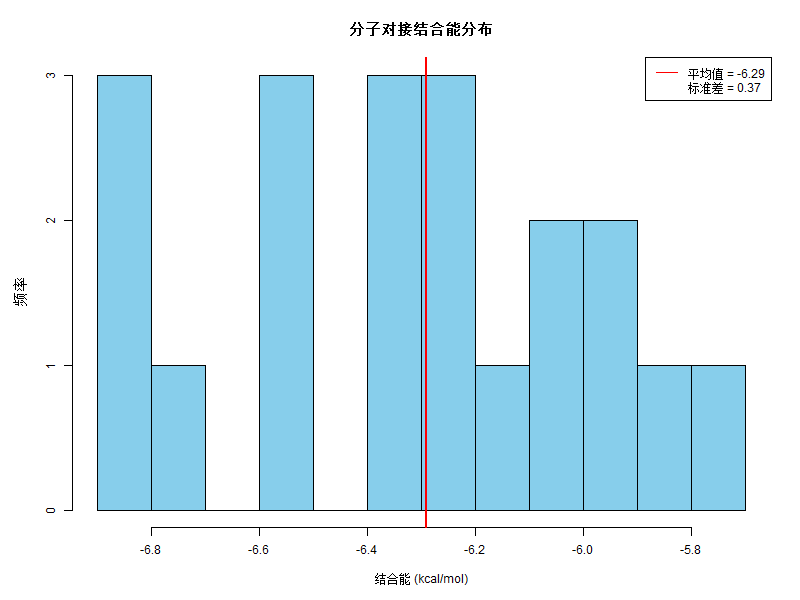

Supplement: S8 Data — (ZIP) [file pone.0332750.s009.zip › 8.Molecular_Docking/对接结果分析/Dihydrochelerythrine-ESR2/docking_energy_distribution.png]

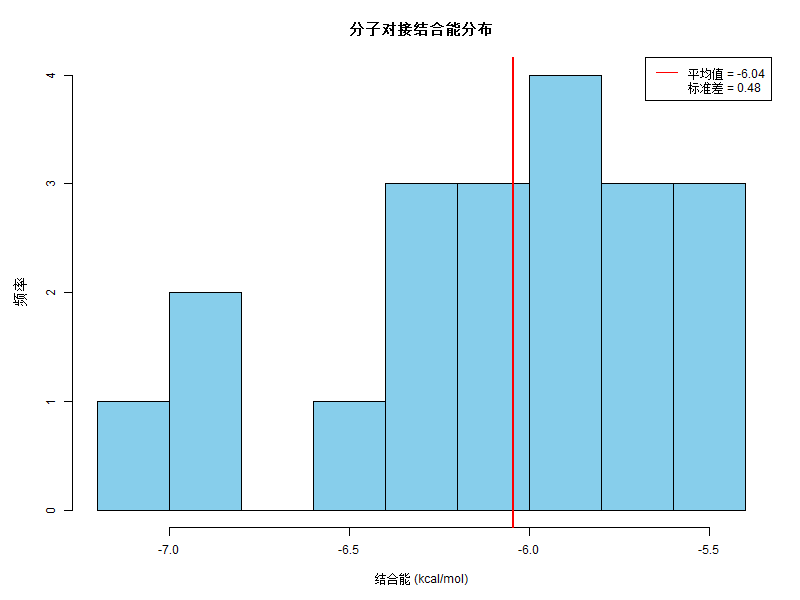

Supplement: S8 Data — (ZIP) [file pone.0332750.s009.zip › 8.Molecular_Docking/对接结果分析/Dihydrochelerythrine-HSP90AA1/docking_energy_distribution.png]

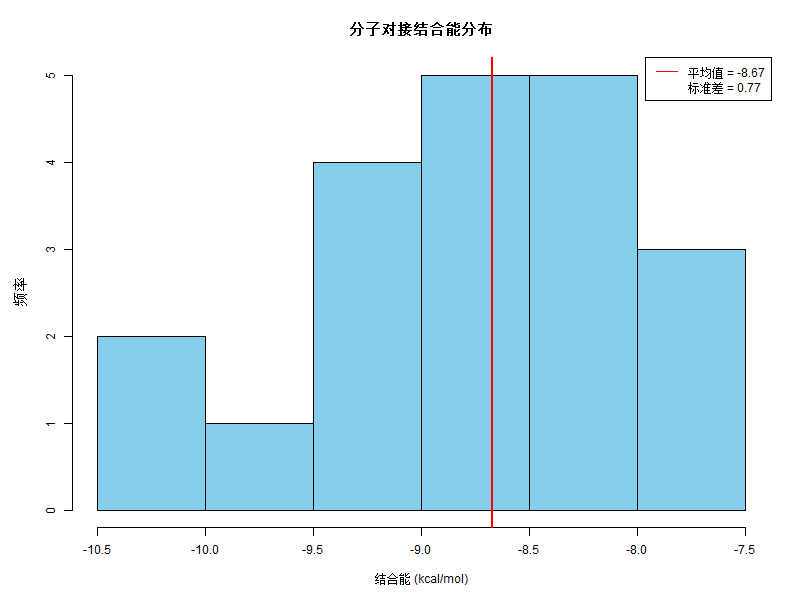

Supplement: S8 Data — (ZIP) [file pone.0332750.s009.zip › 8.Molecular_Docking/对接结果分析/Dihydrochelerythrine-PTGS2/docking_energy_distribution.png]

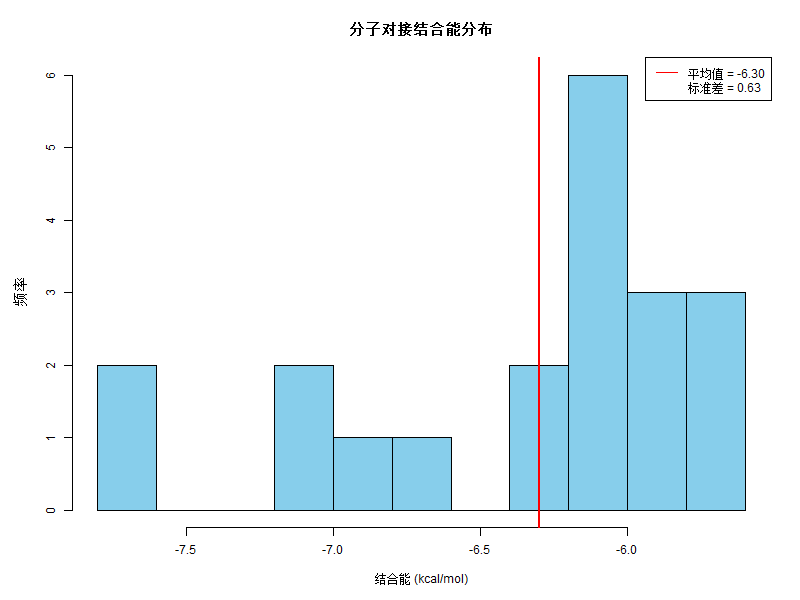

Supplement: S8 Data — (ZIP) [file pone.0332750.s009.zip › 8.Molecular_Docking/对接结果分析/Oxysanguinarine-CALM3/docking_energy_distribution.png]

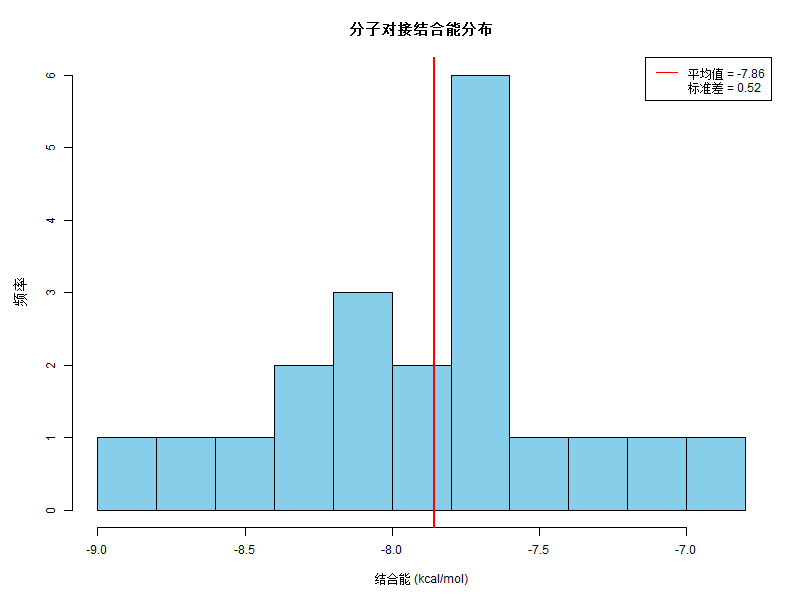

Supplement: S8 Data — (ZIP) [file pone.0332750.s009.zip › 8.Molecular_Docking/对接结果分析/Oxysanguinarine-CASP3/docking_energy_distribution.png]

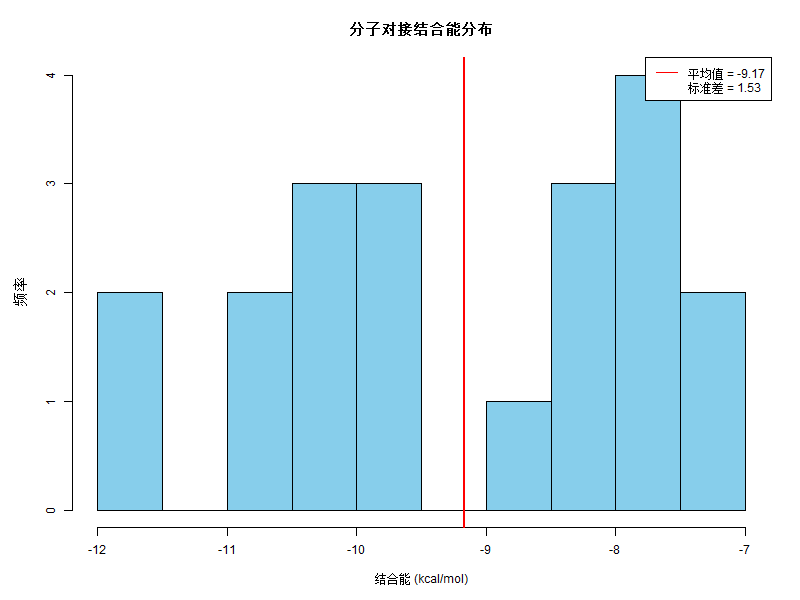

Supplement: S8 Data — (ZIP) [file pone.0332750.s009.zip › 8.Molecular_Docking/对接结果分析/Oxysanguinarine-CDK2/docking_energy_distribution.png]

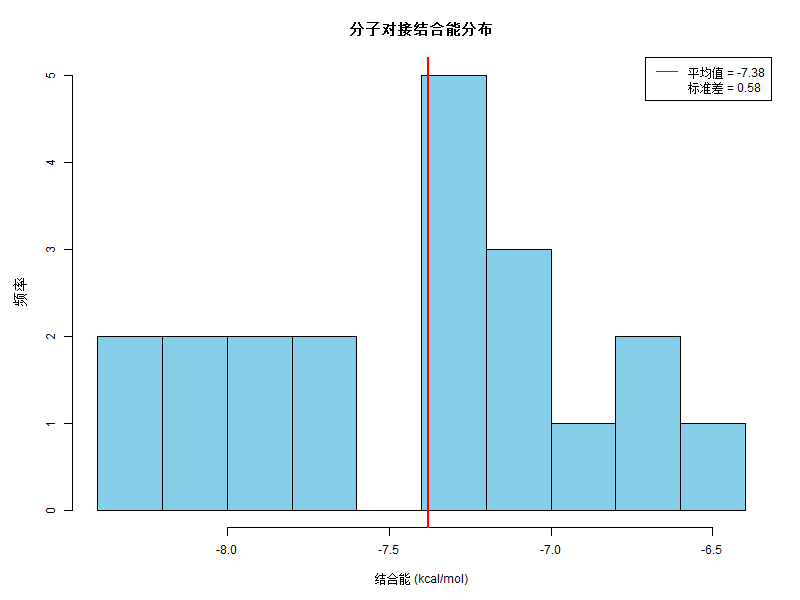

Supplement: S8 Data — (ZIP) [file pone.0332750.s009.zip › 8.Molecular_Docking/对接结果分析/Oxysanguinarine-ESR1/docking_energy_distribution.png]

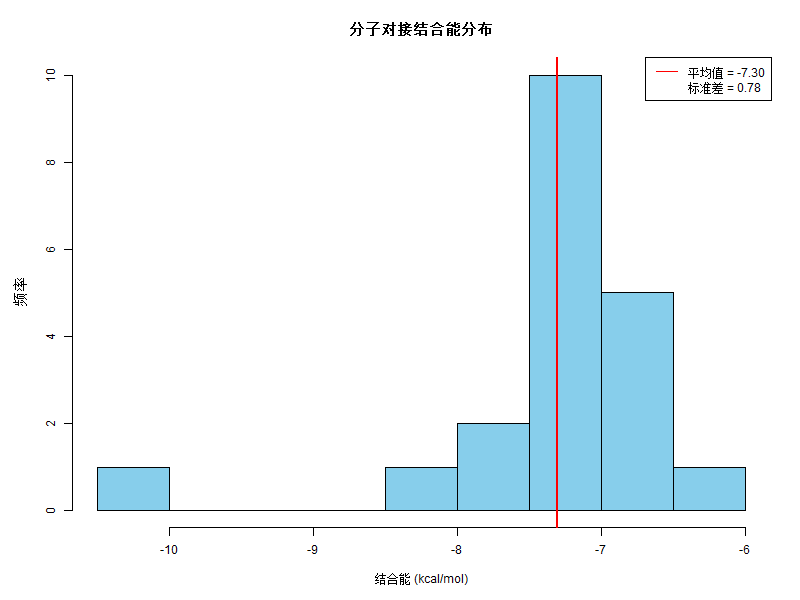

Supplement: S8 Data — (ZIP) [file pone.0332750.s009.zip › 8.Molecular_Docking/对接结果分析/Oxysanguinarine-ESR2/docking_energy_distribution.png]

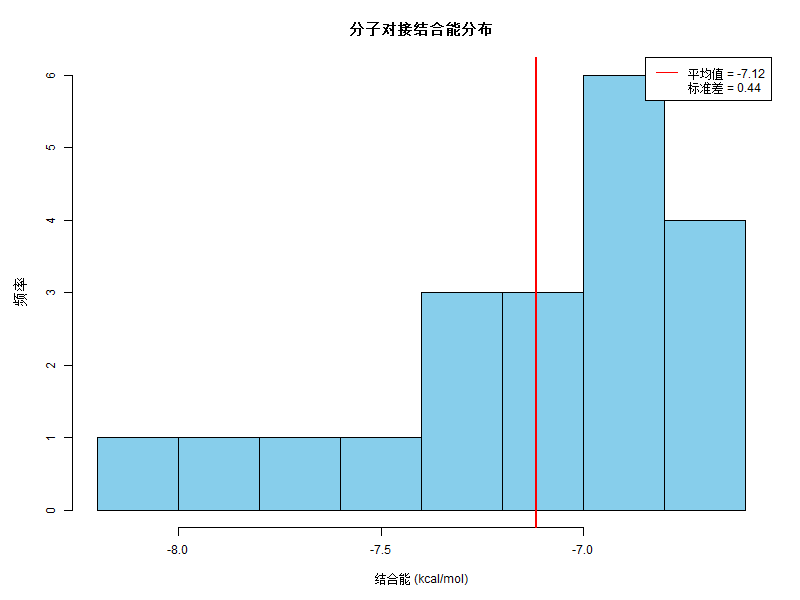

Supplement: S8 Data — (ZIP) [file pone.0332750.s009.zip › 8.Molecular_Docking/对接结果分析/Oxysanguinarine-HSP90AA1/docking_energy_distribution.png]

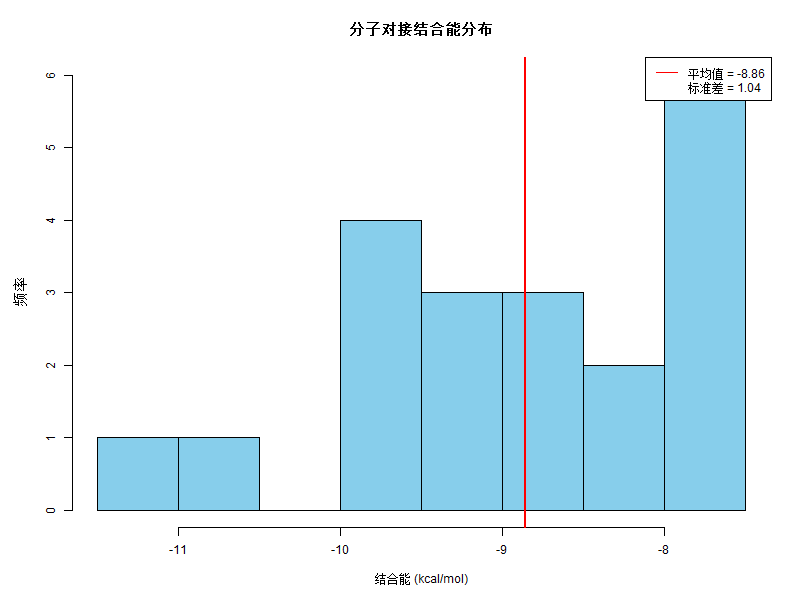

Supplement: S8 Data — (ZIP) [file pone.0332750.s009.zip › 8.Molecular_Docking/对接结果分析/Oxysanguinarine-PTGS2/docking_energy_distribution.png]
